# Supplementary material for: Synthetic microbe-to-plant communication channels
Source: Nat Commun. 2024 Feb 28;15:1817. doi: 10.1038/s41467-024-45897-6 (PMC10901793; doi:10.1038/s41467-024-45897-6)
Supplement: Supplementary file 1 — Supplementary Information [file 41467_2024_45897_MOESM1_ESM.pdf]

Synthetic microbe-to-plant communication channels

Boo and Toth *et al.*

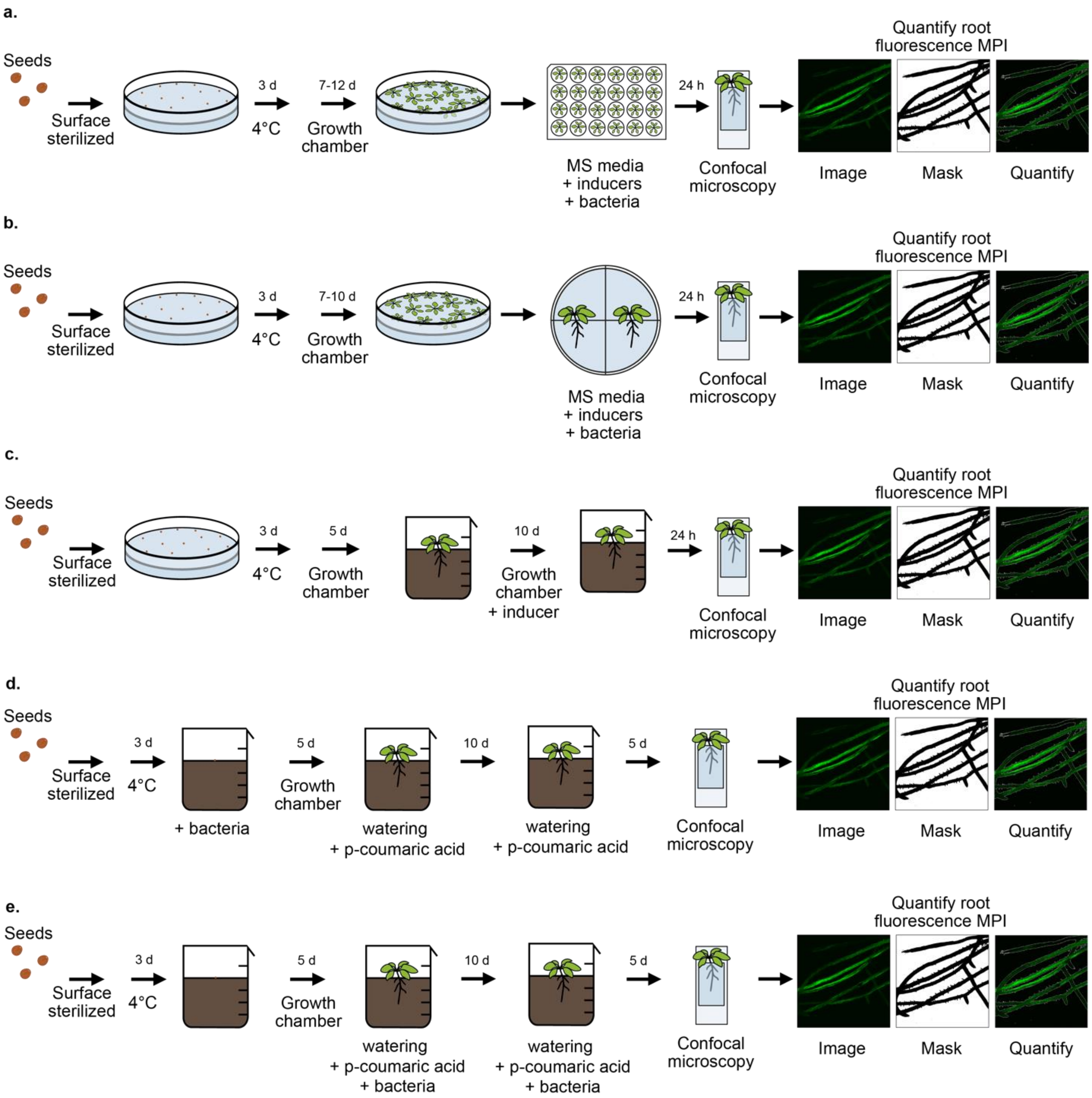

Supplementary Figure 1. *A. thaliana* growth and root imaging workflows. a. The growth of *A. thaliana* in the hydroponic system with chemical inducer (Methods "Chemical HSL induction of A. thaliana HSL receiver in the hydroponic system") or bacteria (Methods "Bacterial induction of *A. thaliana* HSL receiver in the hydroponic system"). This workflow was used for Figures: 2a-d, 3b-d, 4a-e; and Supplementary Figures: 3-6, 8, 10-13, 17-21, 26-29, 31. **b.** The solid media assay with chemical inducer and/or bacteria (Methods "MS agar induction of *A. thaliana* pC-HSL receiver in the hydroponic system"). This workflow was used for Supplementary Figures: 7, 9 and 22. **c.** The chemical induction of *A. thaliana* in sterile soil (Methods "Chemical HSL induction of *A. thaliana* pC-HSL receiver in soil"). This workflow was used for Figures: 2f-g; and Supplementary Figure 14. **d.** The bacterial inoculation of *A. thaliana* seeds in sterile soil (Methods "Bacterial induction of *A. thaliana* pC-HSL receiver by seed inoculation in soil"). This workflow was used for Figures: 3e-f; and Supplementary Figure 23. **e.** The bacteria induction of *A. thaliana* by watering in sterile and non-sterile soil (Methods "Bacterial induction of *A. thaliana* pC-HSL receiver by watering in soil"). This workflow was used to for Figures: 3e-f; and Supplementary Figures 23-24.

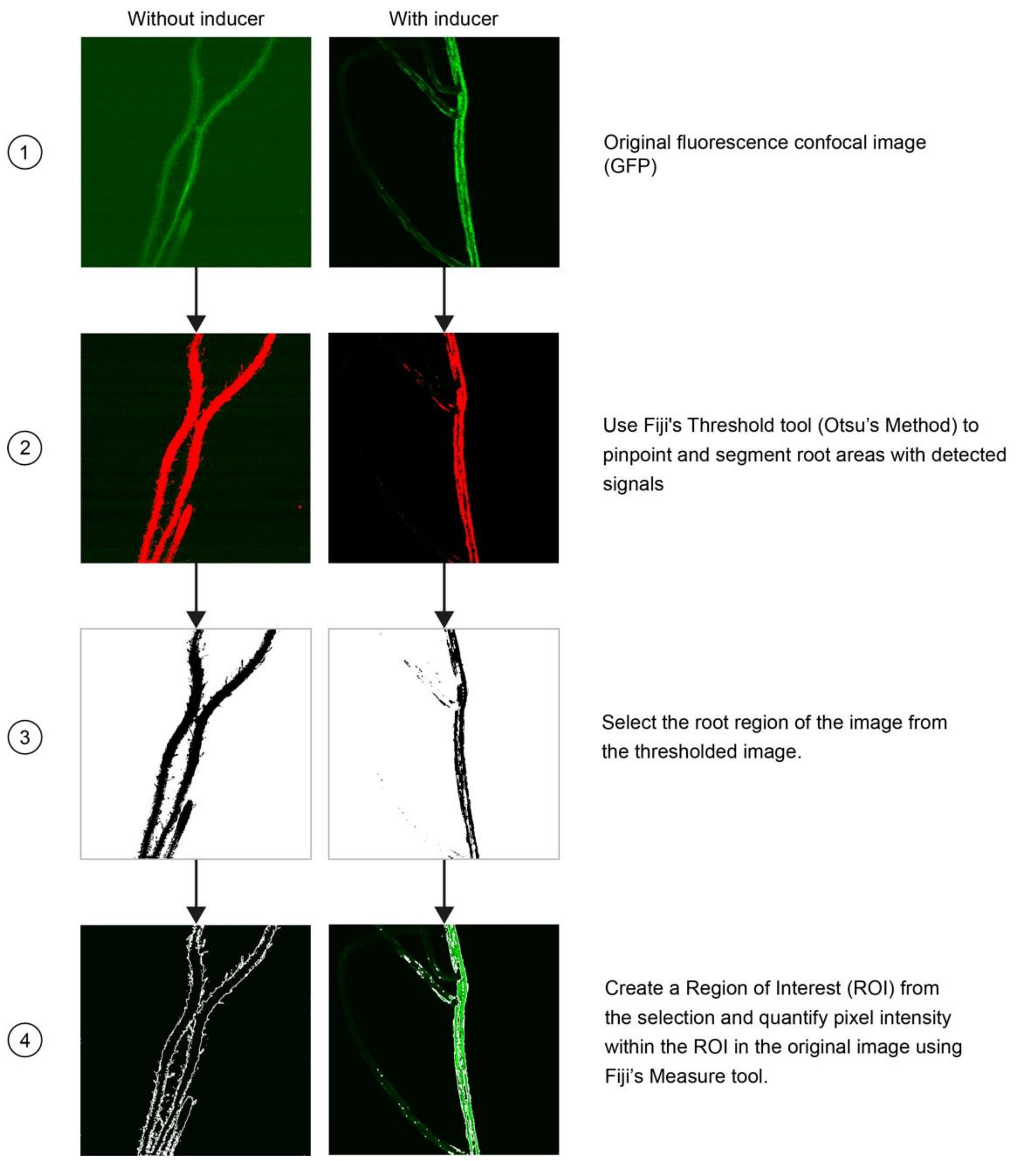

Supplementary Figure 2. Calculation of mean pixel intensity (MPI) from root images. Example images are shown to illustrate the steps used to process images using Fiji to quantify the MPI of root tissue from a fluorescent image (Methods). The Fiji custom script used to perform the MPI calculation is available at Github [https://github.com/VoigtLab/plant-microbe-communication].

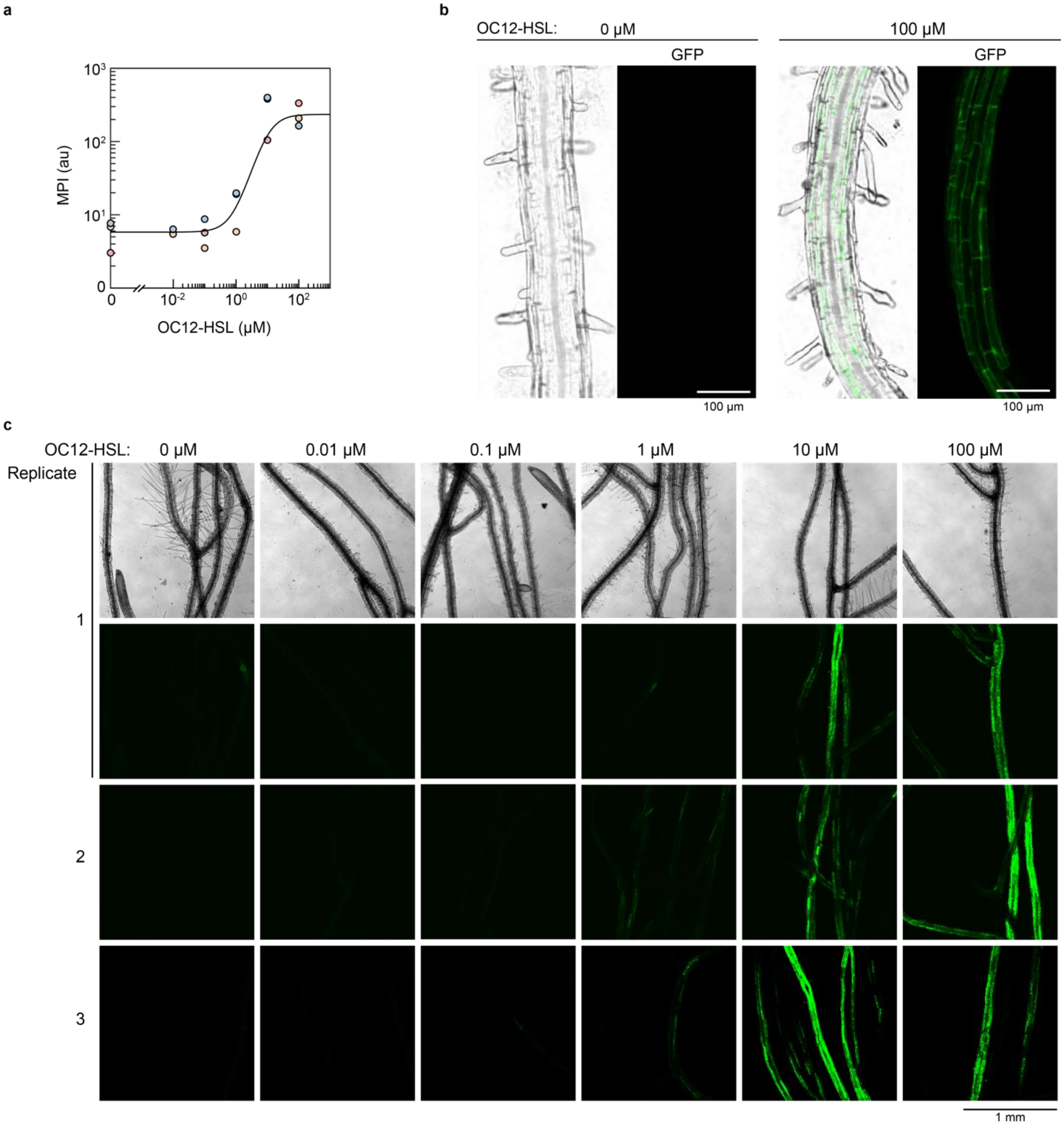

Supplementary Figure 3. The response of the *A. thaliana* OC12-HSL receiver. a. The response function of the *A. thaliana* OC12-HSL receiver is shown (*A. thaliana* 314_12_2). GFP fluorescence increased 40-fold in the presence of 100 μM 3OC12-HSL. The induction was performed for 24 hours in the hydroponic system (Methods). The data represent three experiments performed on different days with different plants. The line is the fit of these data to Equation 1, with the parameters provided in Supplementary Table 1. **b.** Confocal fluorescent images of *A. thaliana* OC12-HSL receiver roots induced in the hydroponic system (Methods). **c.** Root tissue images used to build the response function in part a. Each row represents a replicate performed on different days with different plants. Bright-field and GFP images are provided for the first replicate. Images were taken using the A1R confocal microscope. The experimental protocol is shown in Supplementary Figure 1a and described in Methods. Source data are provided as a Source Data file.

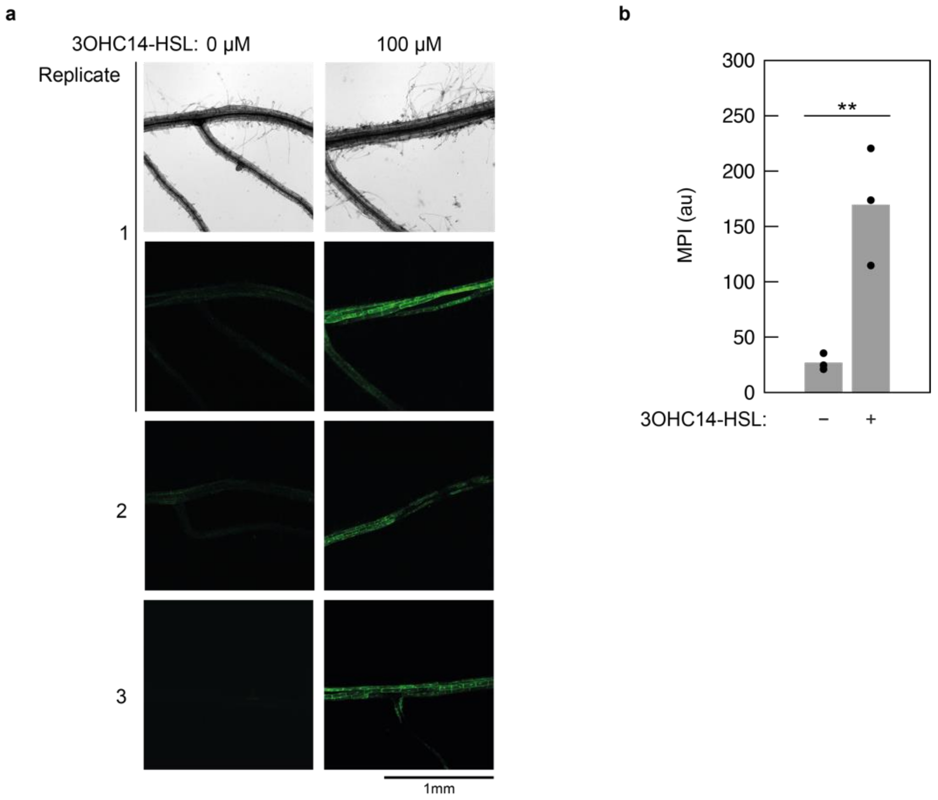

Supplementary Figure 4. The response of the *A. thaliana* OHC14-HSL receiver. a. Confocal fluorescent images of *A. thaliana* OHC14-HSL receiver roots (*A. thaliana* 313_3_4). The induction was performed for 24 hours in the hydroponic system with 0 µM or 100 µM of 3OHC14-HSL (Methods). Each row represents a replicate performed with different plants on a different day. Bright-field and GFP images are provided for the first replicate. Images were taken using the A1R confocal microscope. **b.** The mean fluorescence intensity (MPI) was extracted from the images in a. Data were obtained for $n = 3$ plants on different days and the bars represent the means of these points. The MPI is activated 7-fold in the presence of 100 µM 3OHC14-HSL. The experimental protocol is shown in Supplementary Figure 1a and described in Methods. Statistical significance was determined using two-tailed Student's *t*-test (**, $P < 0.01$). Source data are provided as a Source Data file.

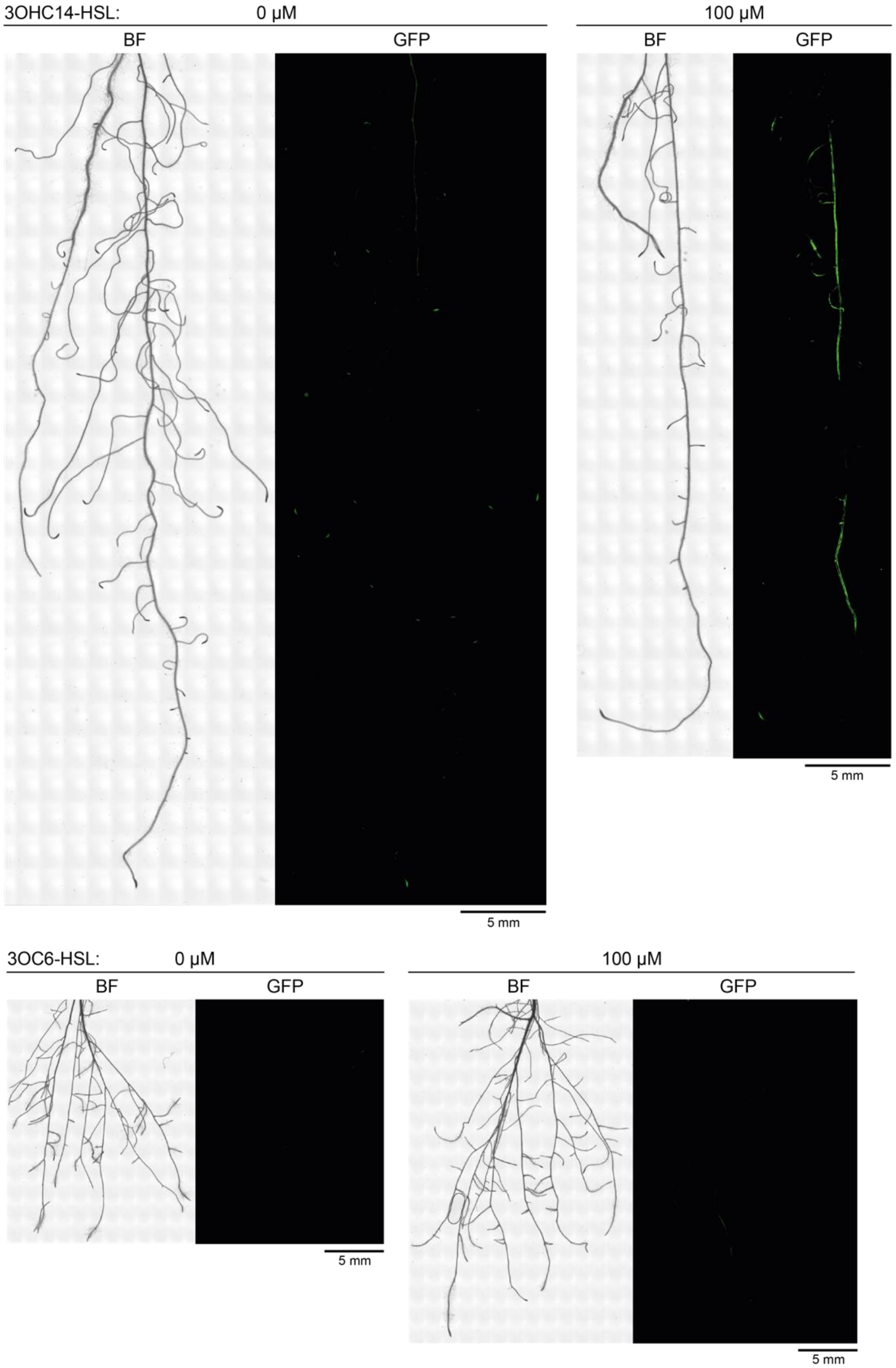

Supplementary Figure 5. Whole-plant imagining of the *A. thaliana* 3OHC14-HSL and 3OC6-HSL receivers. The *A. thaliana* 3OHC14-HSL receiver was induced with 100 µM 3OHC14-HSL (*A. thaliana* 312_7_5). The *A. thaliana* 3OC6-HSL receiver was induced with 100 µM 3OC6-HSL (*A. thaliana* 313_3_4). Whole-root bright-field (BF) and GFP images are shown. Images were taken with the A1R confocal microscope and are representative of experiments performed on three different days with different plants. The experimental protocol is shown in Supplementary Figure 1a and described in Methods.

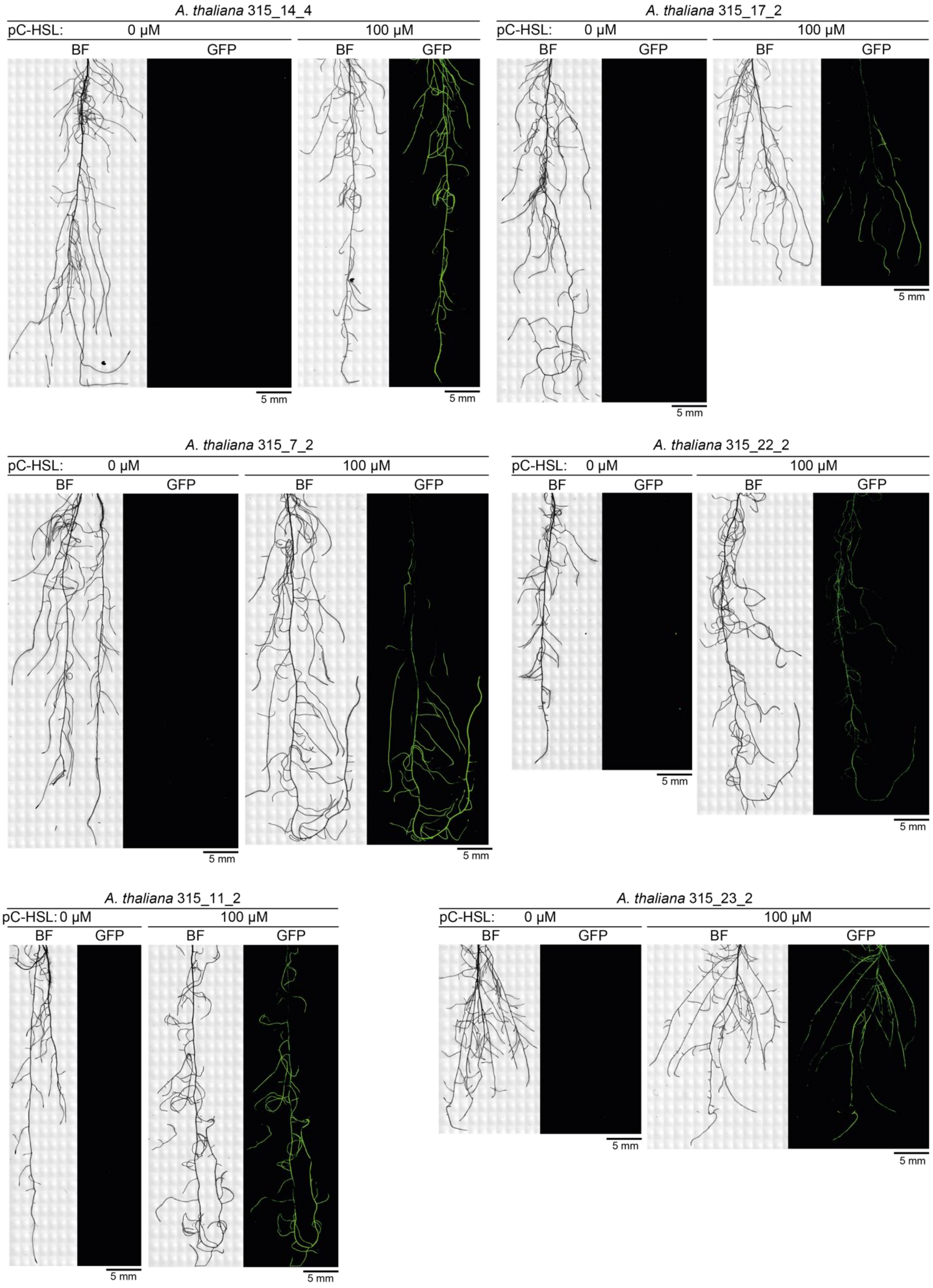
A. thaliana 315_14_4
pC-HSL:
0 µM
100 µM
BF
GFP
BF
GFP
5 mm
5 mm
A. thaliana 315_17_2
pC-HSL:
0 µM
100 µM
BF
GFP
BF
GFP
5 mm
5 mm
A. thaliana 315_7_2
pC-HSL:
0 µM
100 µM
BF
GFP
BF
GFP
5 mm
5 mm
A. thaliana 315_22_2
pC-HSL:
0 µM
100 µM
BF
GFP
BF
GFP
5 mm
5 mm
A. thaliana 315_11_2
pC-HSL: 0 µM
100 µM
BF
GFP
BF
GFP
5 mm
5 mm
A. thaliana 315_23_2
pC-HSL:
0 µM
100 µM
BF
GFP
BF
GFP
5 mm
5 mm

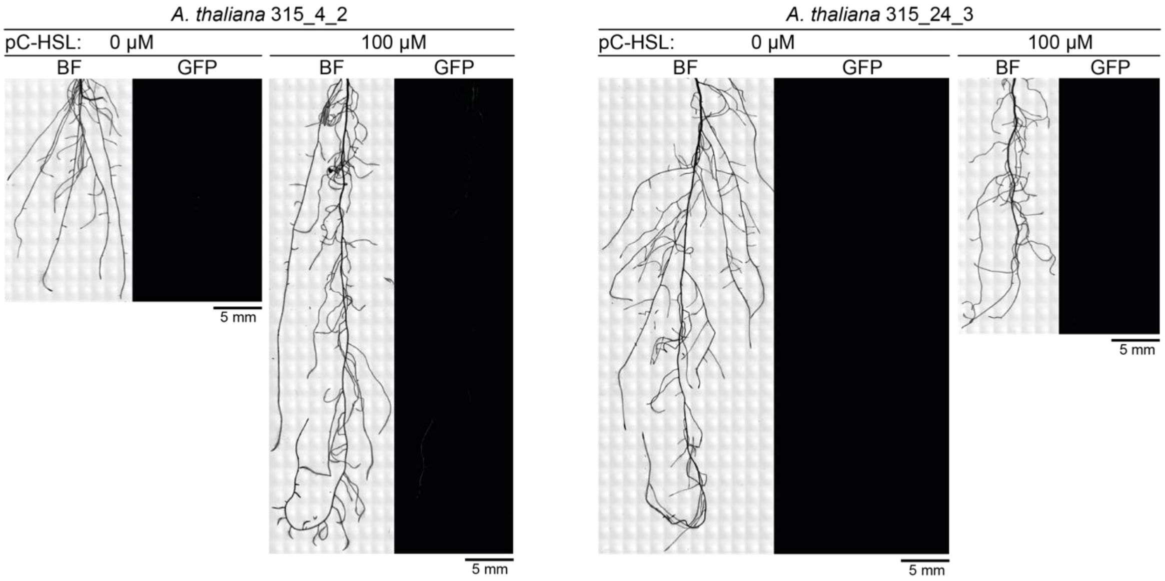

Supplementary Figure 6. Induction of independent lines of *A. thaliana* containing the pC-HSL receiver. Eight independent *A. thaliana* pC-HSL receiver lines were induced with either 0 μM or 100 μM pC-HSL in the hydroponic system for 24 hours (Methods). The *A. thaliana* pC-HSL receiver lines were obtained by transformation of the pTT315 plasmid (Supplementary Figure 30). Whole-root bright-field (BF) and GFP images are shown for each plant line. Images were taken with the A1R confocal microscope. Images are representative of experiments performed on three different days with different plants. The experimental protocol is shown in Supplementary Figure 1a and described in Methods.

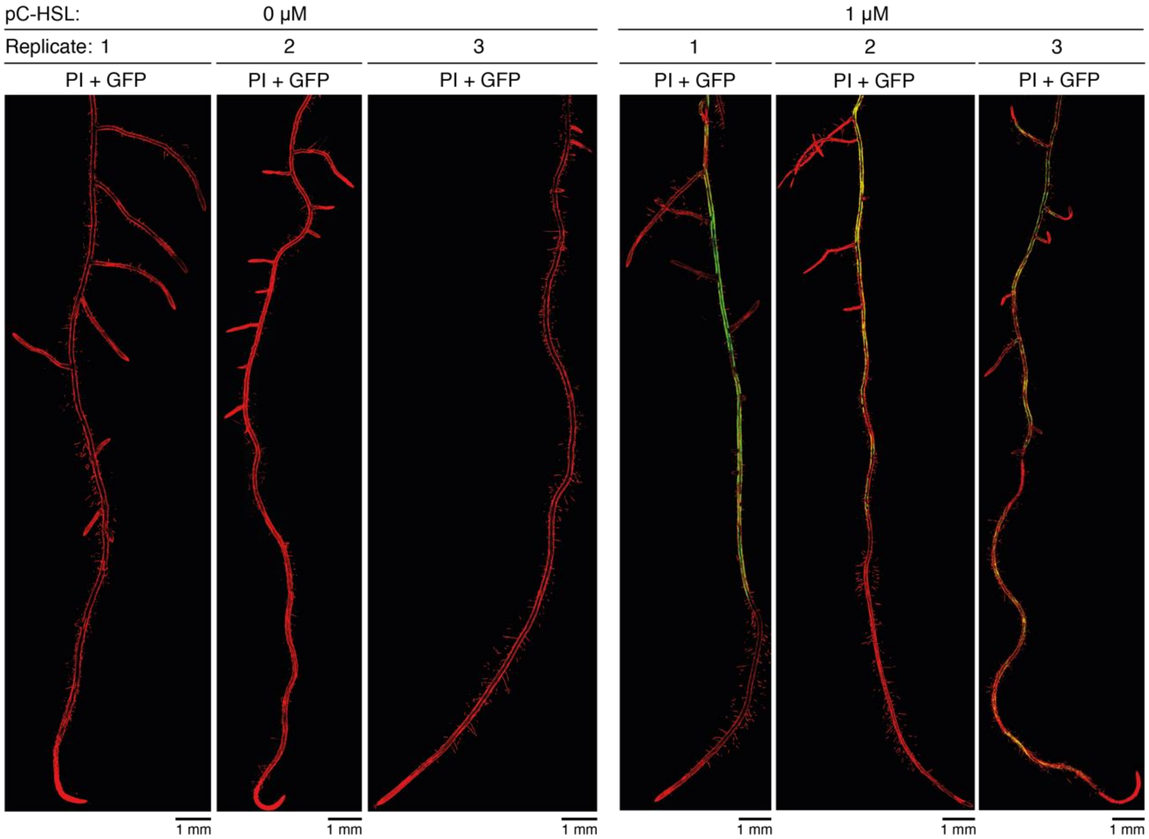

Supplementary Figure 7. Spatiotemporal characterization of the induction of the *A. thaliana* pC-HSL receiver by pC-HSL in the hydroponic system. The *A. thaliana* pC-HSL receiver was induced with either 0 µM or 100 µM pC-HSL for 24 hours in the hydroponic system (*A. thaliana* 315_14_5_1). Cell wall staining was achieved using propidium iodide (PI), and whole-root images display the composite images for PI and GFP. Each replicate corresponds to a different plant imaged on a different day (*n* = 3). Images were taken with the Leica SP8 confocal microscope. The experimental protocol is shown in Supplementary Figure 1a and described in Methods.

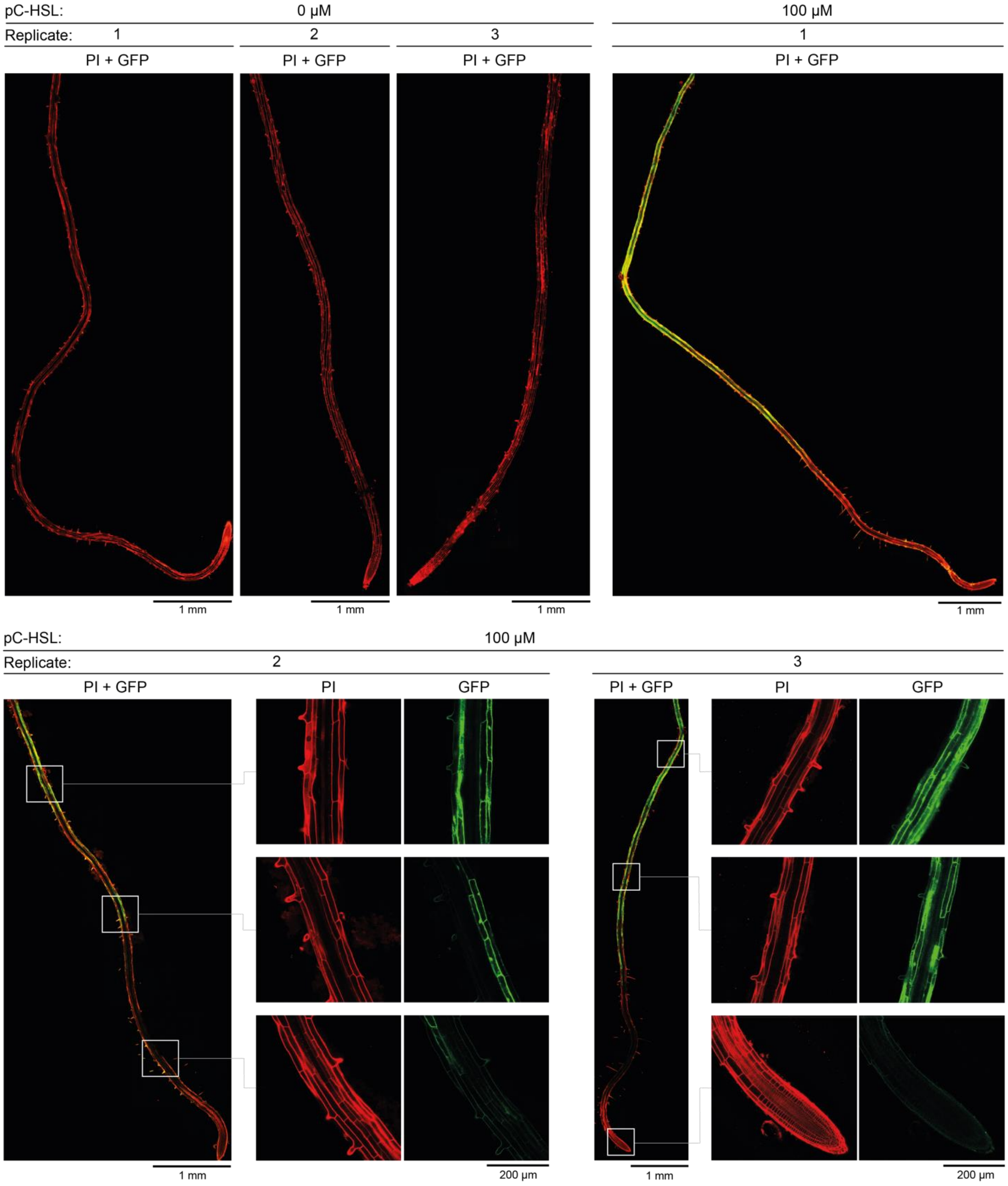

Supplementary Figure 8. Spatiotemporal characterization of the induction of the *A. thaliana* pC-HSL receiver by pC-HSL on solid media. The *A. thaliana* pC-HSL receiver was induced with either 0 µM or 100 µM pC-HSL on MS agar plates for 24 hours (*A. thaliana* 315_14_5_1). Cell wall staining was achieved using propidium iodide (PI), and whole-root images display the composite images for PI and GFP. Each replicate corresponds to a different plant imaged on a different day (n = 3). Images were taken with the Leica SP8 confocal microscope. The experimental protocol is shown in Supplementary Figure 1b and described in Methods.

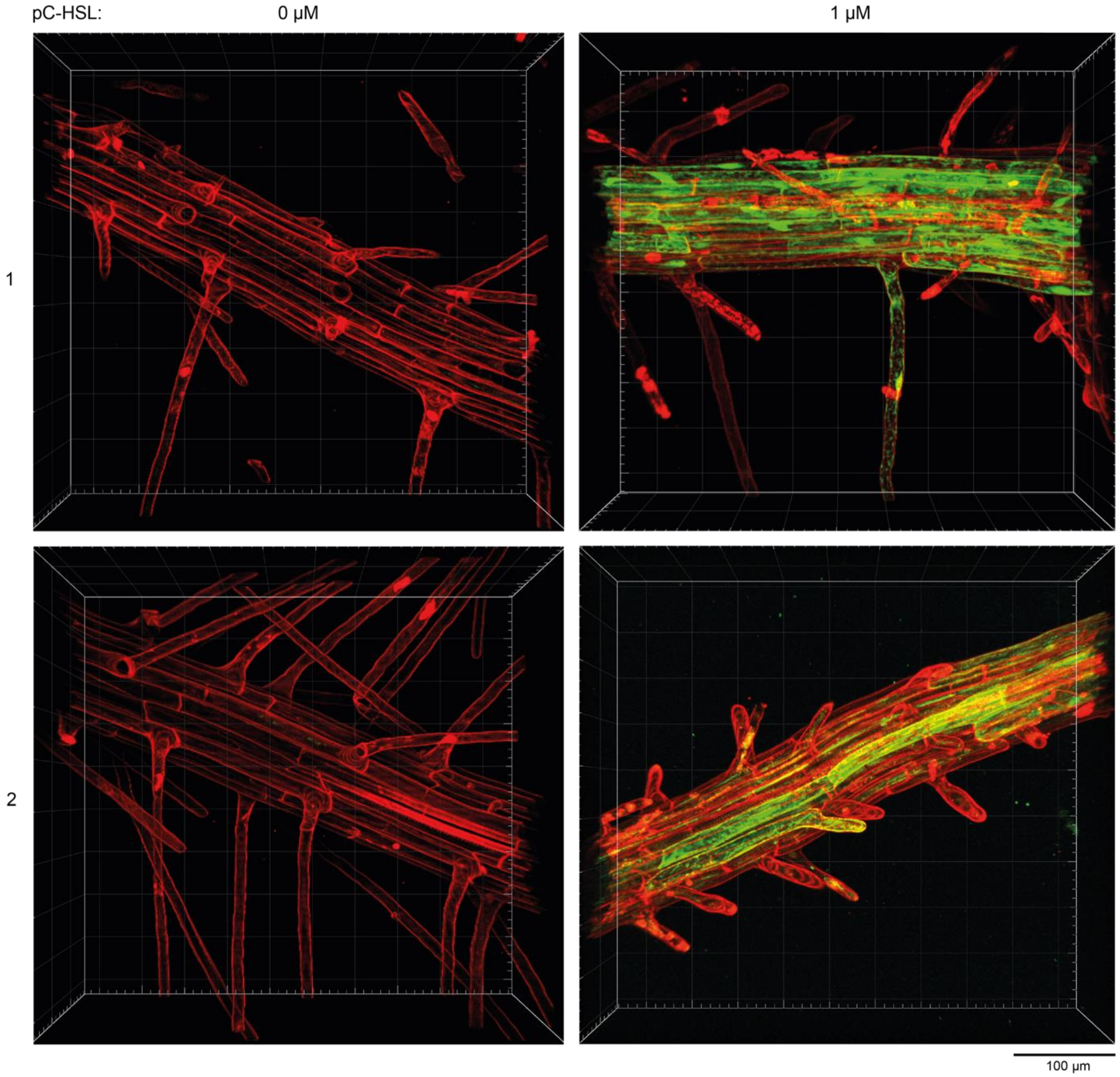

Supplementary Figure 9. Root hair induction of the *A. thaliana* pC-HSL receiver by pC-HSL in the hydroponic system. The *A. thaliana* pC-HSL receiver was induced with either 0 μM or 1 μM pC-HSL for 24 hours in the hydroponic system (*A. thaliana* 315_14_5_1). Cell wall staining was achieved using propidium iodide (PI), and whole-root images display the composite images for PI and GFP. Each replicate corresponds to a different plant imaged on a different day (n = 2). Images were taken with the Leica SP8 confocal microscope. The experimental protocol is shown in Supplementary Figure 1a and described in Methods. Videos of the 3D reconstruction for all images are available as Supplementary Movies 1-4.

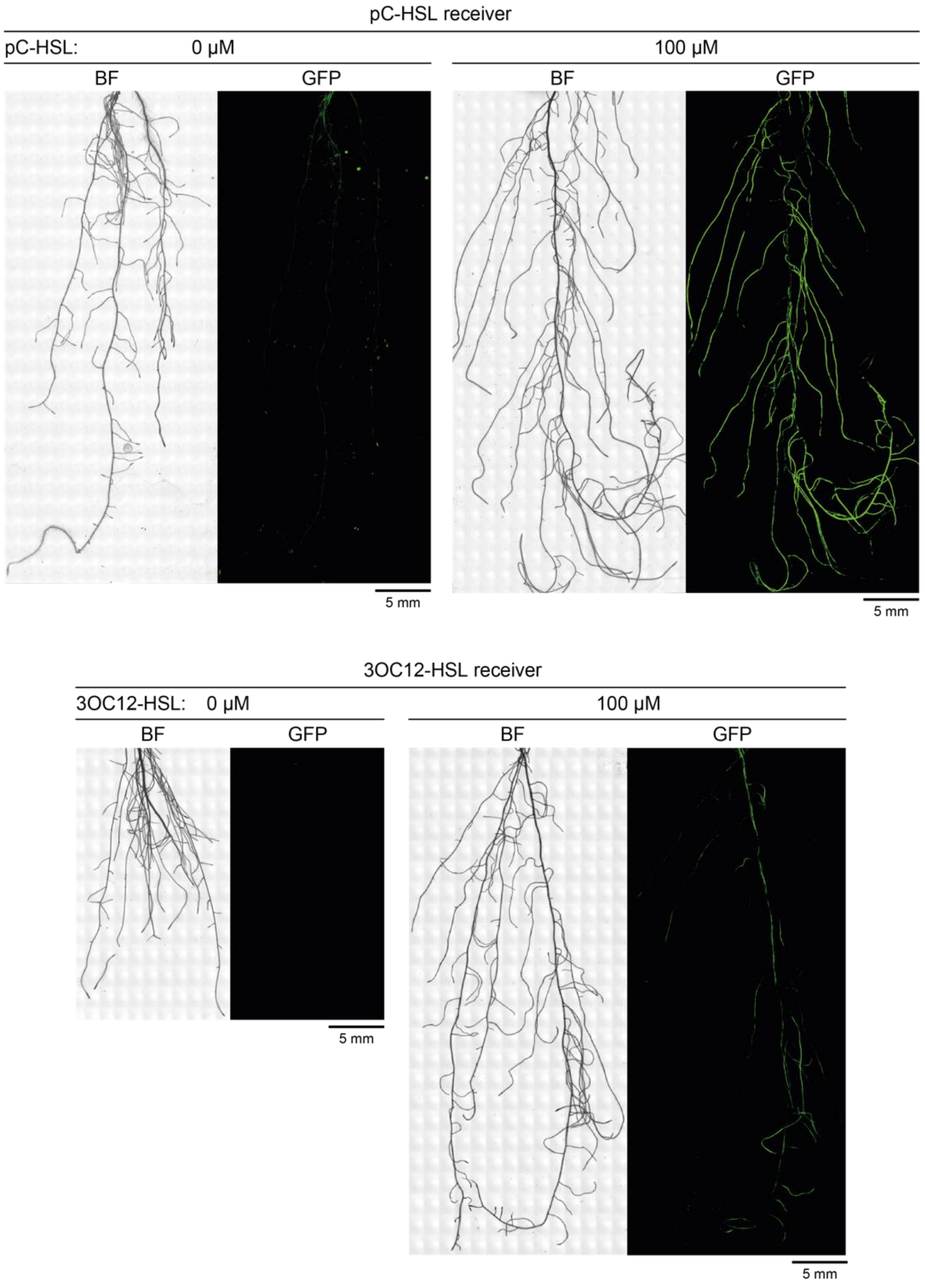

Supplementary Figure 10. Whole-root imagining of the *A. thaliana* pC-HSL and 3OC12-HSL receivers. Chemical induction in the hydroponic system (Methods) of the pC-HSL and 3OC12-HSL receiver plant lines with 0 µM and 100 µM of their respective HSL (*A. thaliana* 315_14_5_1 and *A. thaliana* 314_12_2). Whole-root bright-field (BF) and GFP images are shown for each plant line. Images were taken with the A1R confocal microscope (Methods) and are representative of experiments performed on three different days with different plants. The experimental protocol is shown in Supplementary Figure 1a and described in Methods.

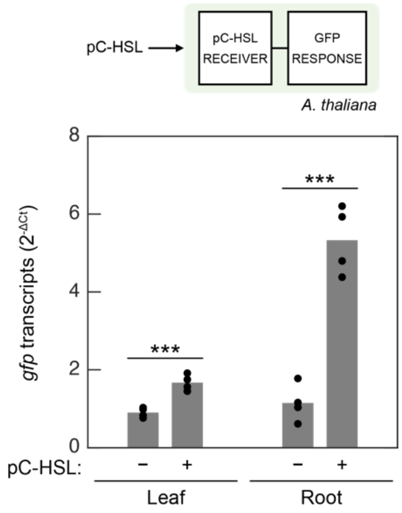

Supplementary Figure 11. Induction of the pC-HSL receiver in the *A. thaliana* roots and leaves. The *A. thaliana* pC-HSL receiver was induced with 0 µM (-) and 100 µM (+) pC-HSL on MS agar. qRT-PCR was used to measure the induction of *gfp* (Methods) (*A. thaliana* 315_14_5_1). Data points represent the *gfp* transcripts for four plants grown on different days. The expression of *gfp* transcripts exhibited a 2-fold increase in leaves and a 5-fold increase in roots (fold-change defined as the ratio of 100 µM pC-HSL to 0 µM). Statistical significance was determined using two-tailed Student's *t*-test (***, $P < 0.001$). Source data are provided as a Source Data file.

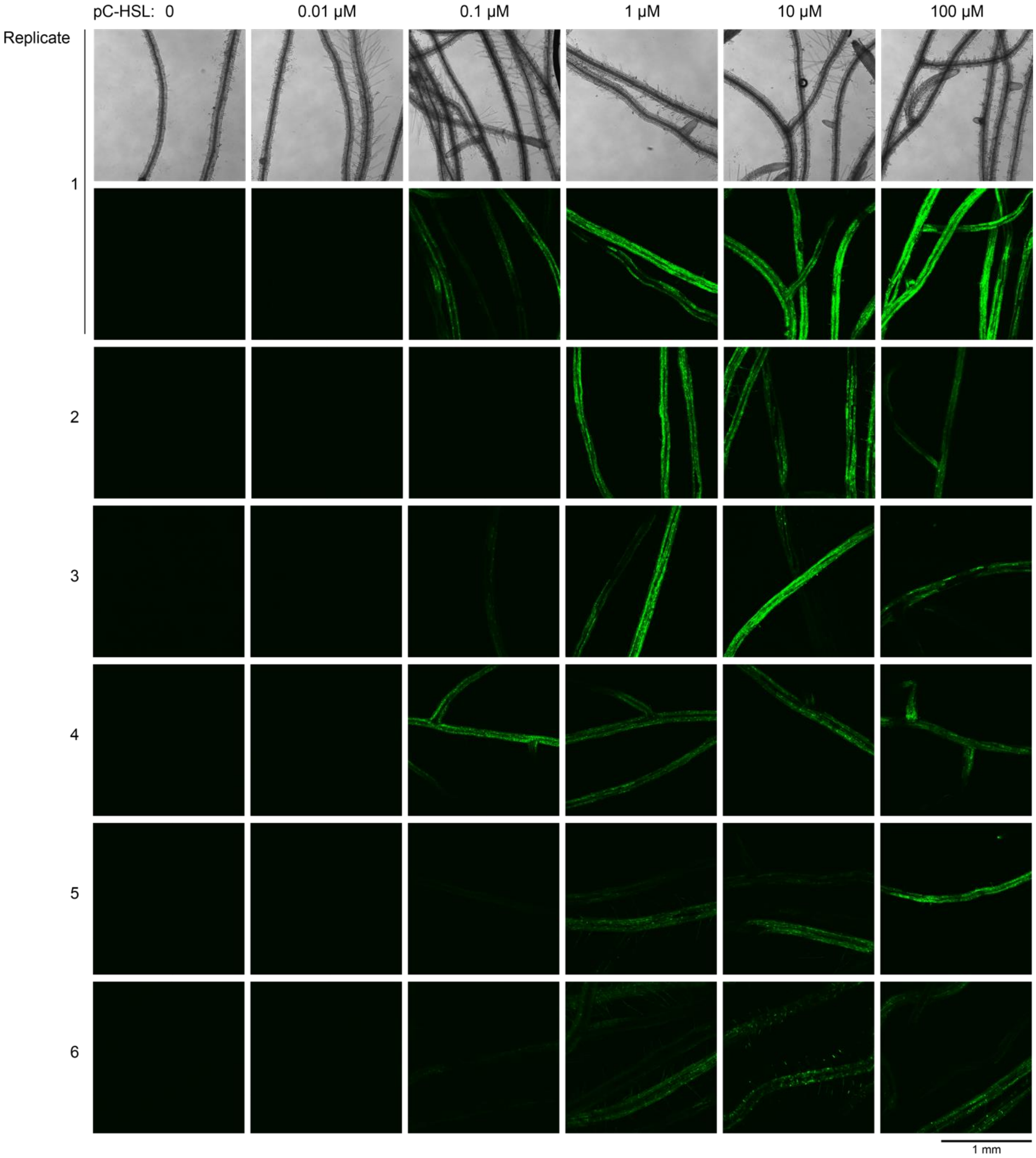

Supplementary Figure 12. Images used to calculate the response function of the *A. thaliana* pC-HSL receiver. The raw images of root tissue used to generate the data in Figure 2c, including statistics. Each row represents a replicate performed on different days with different plants (*A. thaliana* 315_14_5). Bright-field (BF) images are provided for the first replicate. Images were taken using the A1R confocal microscope. The experimental protocol is shown in Supplementary Figure 1a and described in Methods.

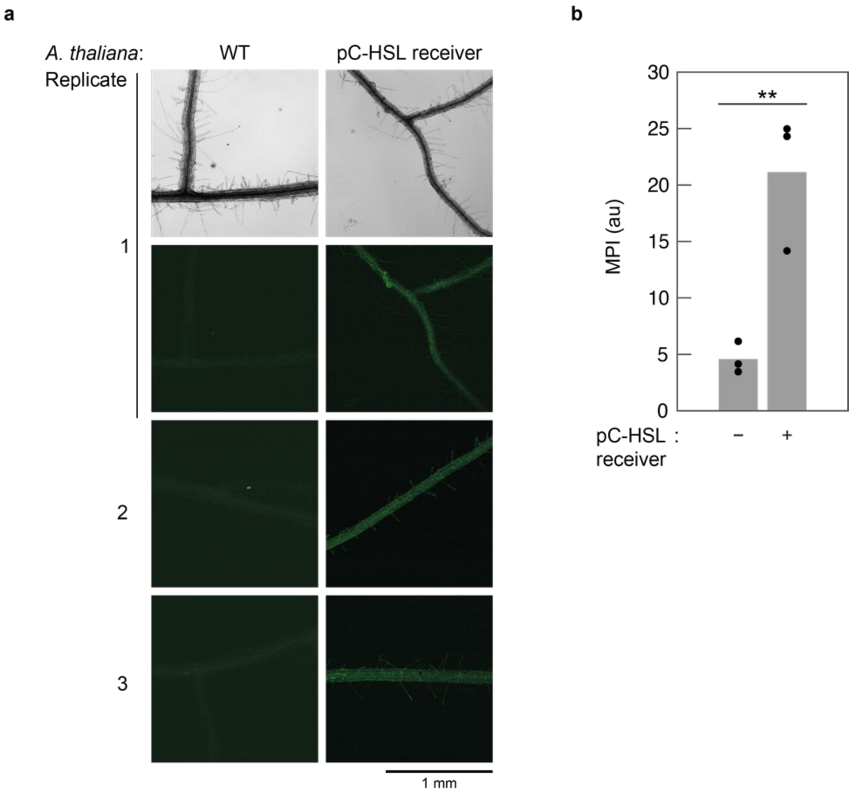

Supplementary Figure 13. Comparison of the background fluorescence of *A. thaliana* with and without the pC-HSL receiver. a. Microscopy images of the root tissue of *A. thaliana* with and without the pC-HSL receiver (*A. thaliana* 15_14_5_1 and Col-0). Each row represents a replicate performed with plants grown for 24 hours in the hydroponic system (Methods). Images were taken using the A1R confocal microscope. The experimental protocol is shown in Supplementary Figure 1a and described in Methods. **b.** Mean pixel intensity (MPI) was calculated from images in panel a. Data were obtained for $n = 3$ plants on different days from panel a and the bars are their means. Statistical significance was determined using two-tailed Student's *t*-test (**, $P < 0.01$). Source data are provided as a Source Data file.

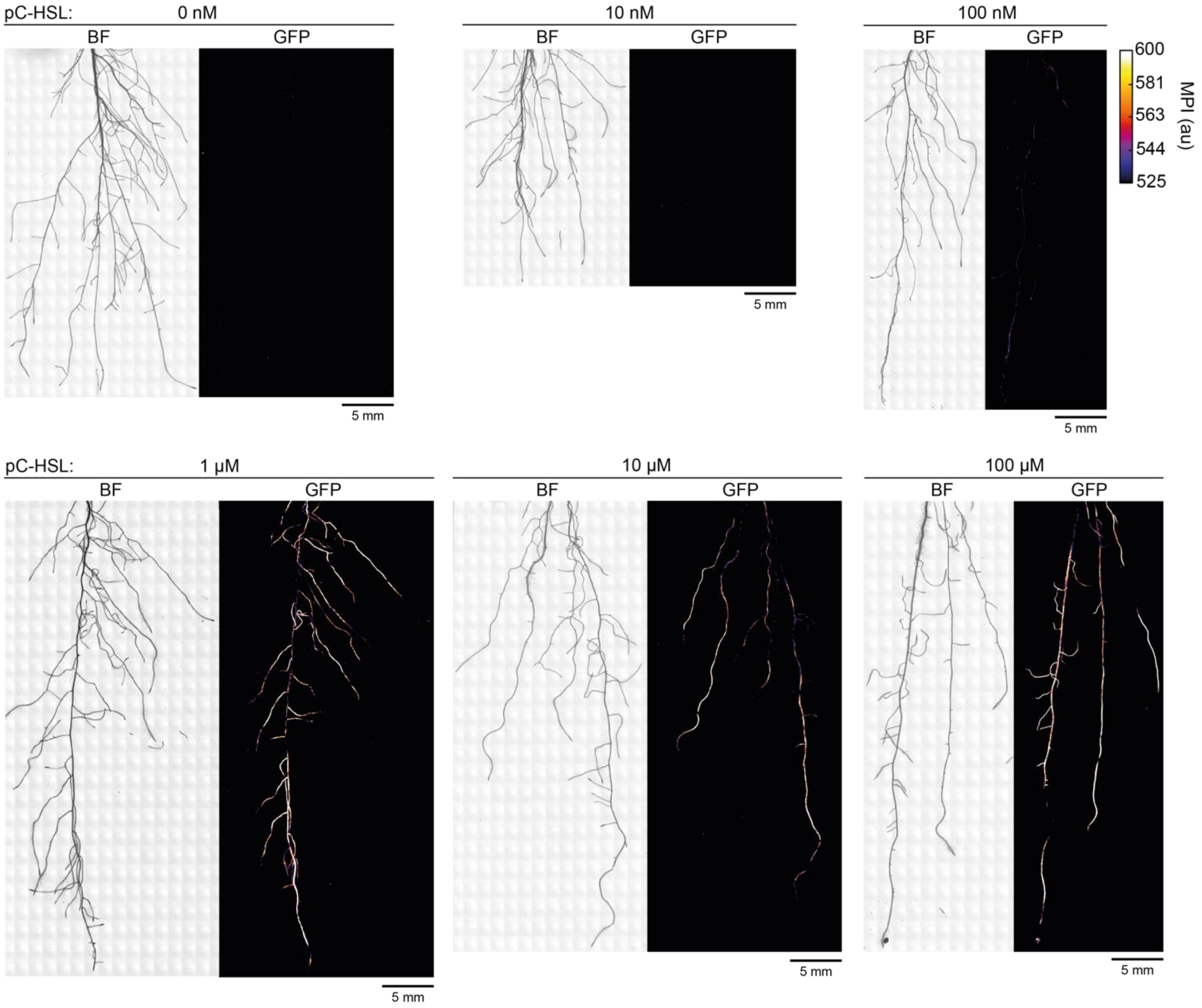

Supplementary Figure 14. Whole-root imaging of the *A. thaliana* pC-HSL receiver titrating pC-HSL. The *A. thaliana* pC-HSL receiver was induced for 24 hours in the hydroponic system with different concentrations of pC-HSL and imaged using the A1R confocal microscope (*A. thaliana* 315_14_5_1, Supplementary Table 3). Whole-root bright-field (BF) and GFP images are shown for each concentration. Images are representative of experiments performed on three different days with different plants. The experimental protocol is shown in Supplementary Figure 1a and described in Methods.

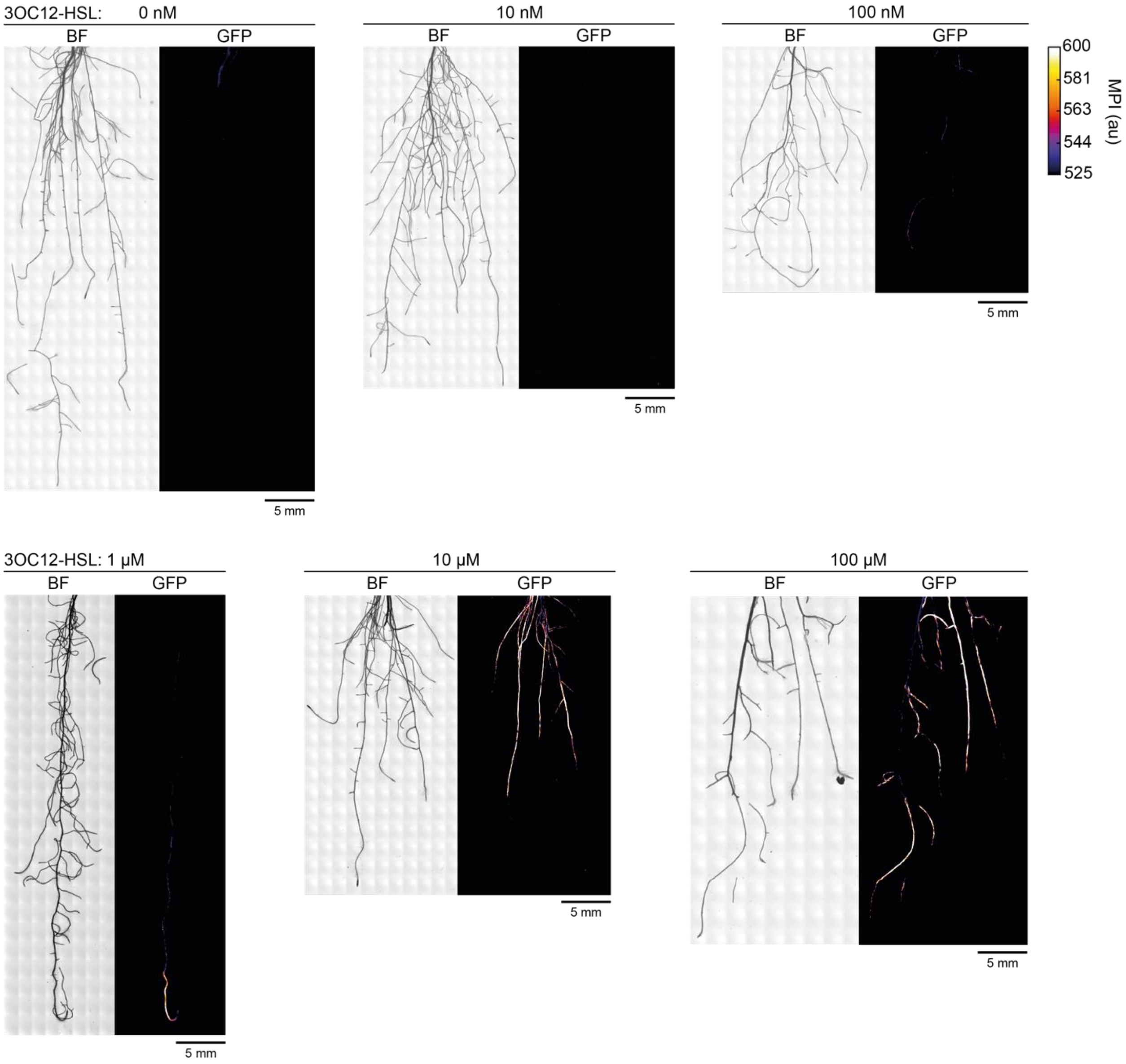

Supplementary Figure 15. Whole-root imaging of the *A. thaliana* 3OC12-HSL receiver titrating 3OC12-HSL. The *A. thaliana* 3OC12-HSL receiver was induced for 24 hours in the hydroponic system with different concentrations of pC-HSL and imaged using the A1R confocal microscope (*A. thaliana* 314_12_2). Whole-root bright-field (BF) and GFP images are shown for each concentration. Images are representative of experiments performed on three different days with different plants. The experimental protocol is shown in Supplementary Figure 1a and described in Methods.

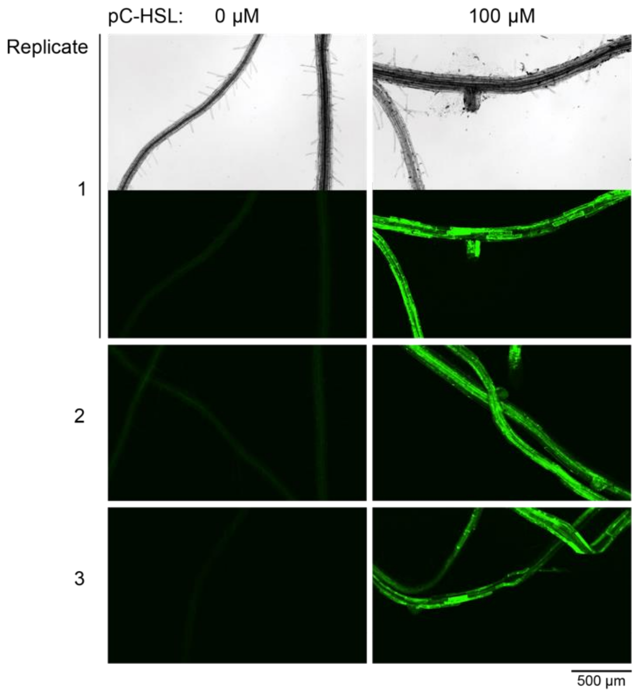

Supplementary Figure 16. Images of the *A. thaliana* pC-HSL receiver induced in soil. The raw images of root tissue used to generate the data in Figure 2g, including statistics. The experimental details are provided in Methods. Each row represents a replicate performed on different days with different plants (*A. thaliana* 315_14_5_1). Images were taken with the A1R confocal microscope. Bright-field and GFP images are provided for the first replicate. The experimental protocol is shown in Supplementary Figure 1c and described in Methods.

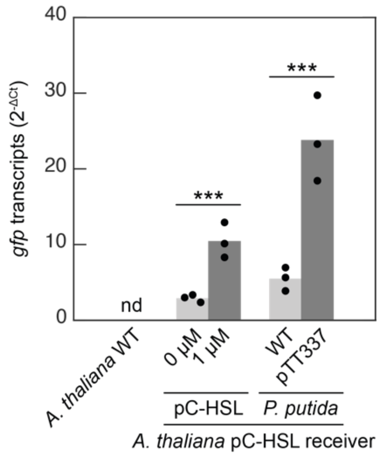

Supplementary Figure 17. Comparison of the induction of the *A. thaliana* receiver by exogenous and microbially-produced pC-HSL. *A. thaliana* wild-type was induced with 0 µM pC-HSL in the hydroponic system in MS medium (with 100 µM *p*-coumarate). The *A. thaliana* pC-HSL receiver (315_14_5_1) was induced with exogenous pC-HSL or incubated with *P. putida* WT or *P. putida* pTT337 to produce pC-HSL in the hydroponic system in MS medium supplemented with 100 µM *p*-coumarate. qRT-PCR was used to measure the induction of *gfp* (Methods). Data points represent the *gfp* transcripts for $n = 3$ plants grown on different days. nd indicates that no *gfp* transcripts were detected. The *gfp* transcripts were upregulated 4-fold when inducing between 0 µM and 1 µM; and 4-fold when inducing between *P. putida* WT and *P. putida* pTT337. Statistical significance was determined using two-tailed Student's *t*-test (***, $P < 0.001$). Source data are provided as a Source Data file.

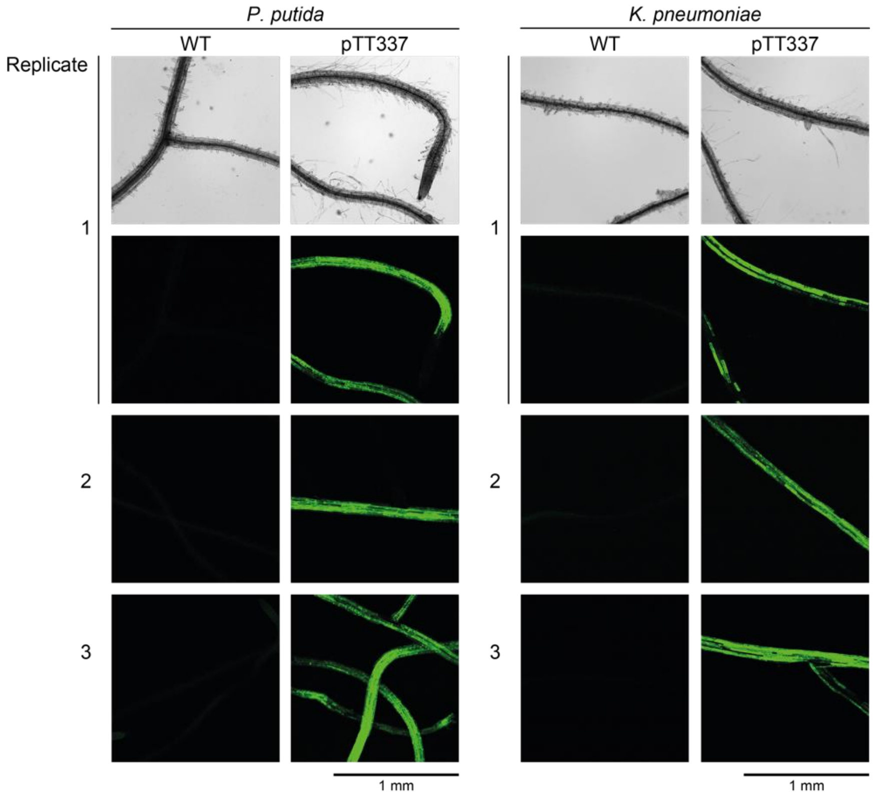

Supplementary Figure 18. The response of the *A. thaliana* pC-HSL receiver to the *P. putida* and *K. pneumoniae* pC-HSL senders. The raw images of root tissue used to generate the data in Figure 3c. The experimental details are provided in Methods. Each row represents a replicate performed on different days with different plants (*A. thaliana* 315_14_5_1). Images were taken with the A1R confocal microscope. Bright-field (BF) and GFP images are provided for the first replicate. The experimental protocol is shown in Supplementary Figure 1a and described in Methods.

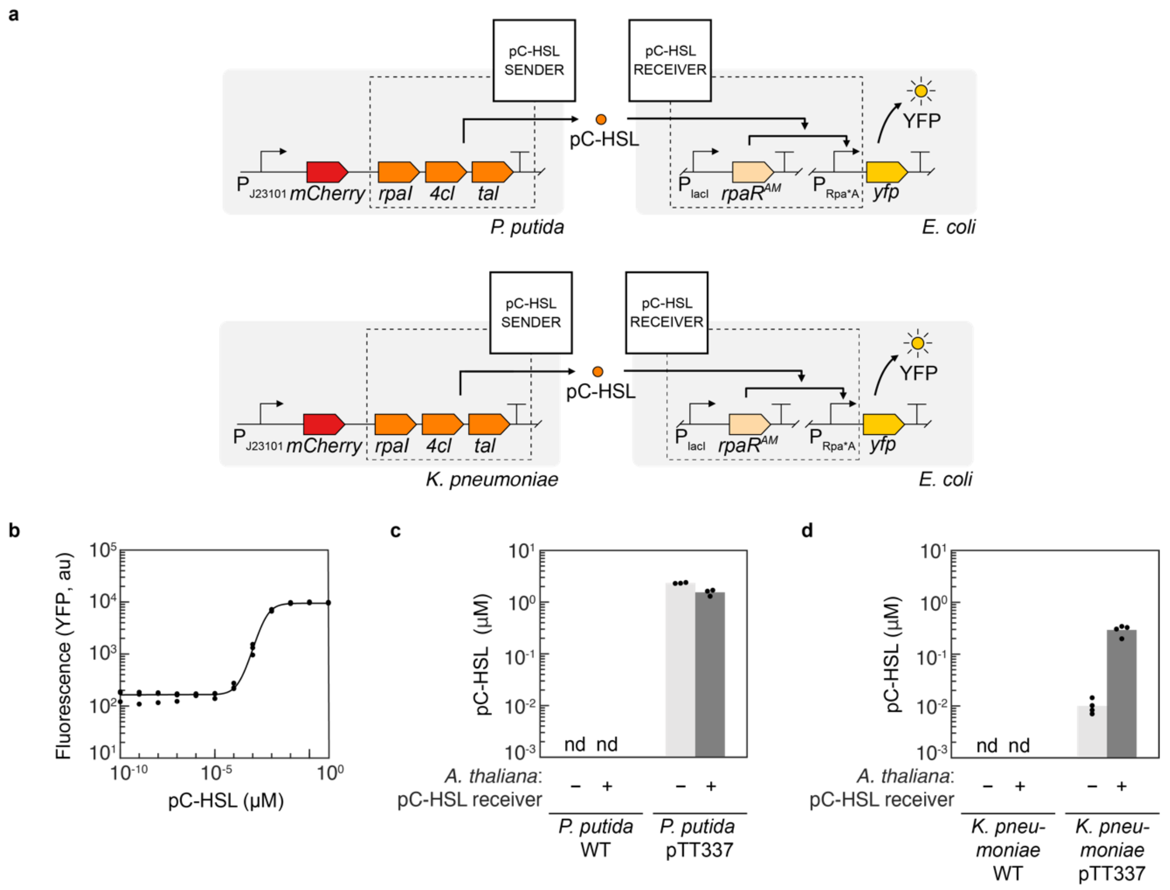

Supplementary Figure 19. Quantification of pC-HSL concentration when produced by engineered *P. putida* and *K. pneumoniae*. a. The constitutive production of pC-HSL by *P. putida* and *K. pneumoniae* was used to induce the *E. coli* pC-HSL receiver (*E. coli* MG1655 sTT658). **b.** The response function of the *E. coli* pC-HSL receiver. Data points were obtained for n = 3 experiments on different days. The line is the fit to Equation 1 (Supplementary Table 2). **c.** The estimated concentration of pC-HSL in the supernatant produced by *P. putida* pTT337 grown for 24 hours with or without the *A. thaliana* pC-HSL receiver (Methods). The *P. putida* strains (*P. putida* wild-type and *P. putida* carrying pTT337, Supplementary Figure 34) were grown in MS medium supplemented with 100 μM p-coumarate. The pC-HSL concentration was calculated by regression using the *E. coli* pC-HSL receiver strain. Data points were obtained for n = 3 experiments on different days and the bars represent the means. nd indicates that no pC-HSL was detected. **d.** The calculated concentration of pC-HSL in the supernatant produced from *K. pneumoniae* carrying pTT337 grown for 24 hours with or without the *A. thaliana* pC-HSL receiver. The *K. pneumoniae* strains (*K. pneumoniae* wild-type and *K. pneumoniae* carrying pTT337) were grown in MS medium supplemented with 100 μM p-coumarate. The pC-HSL concentration was calculated by regression using the *E. coli* pC-HSL receiver strain. Data points were obtained for n = 3 experiments on different days and the bars represent the means of these points. nd indicates that no pC-HSL was detected. Source data are provided as a Source Data file.

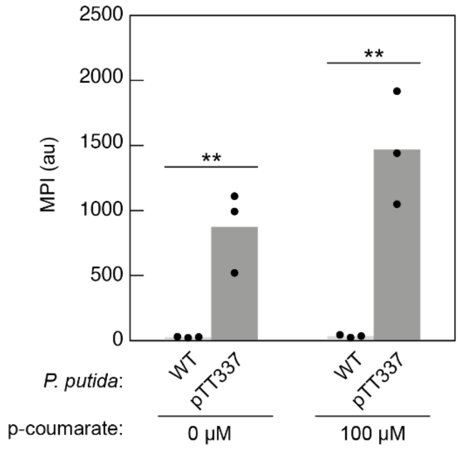

Supplementary Figure 20. Induction of *A. thaliana* pC-HSL receiver with *P. putida* producing pC-HSL. The mean pixel intensity (MPI) of the *A. thaliana* pC-HSL receiver (*A. thaliana* 315_14_5_1) grown in the hydroponic system for 24 hours (Methods). Data points were obtained for $n = 3$ plants on different days and the bars represent the means of these points. Mean pixel intensity (MPI) was measured from images taken with the A1R confocal microscope. Without p-coumarate, there is a 33-fold induction between *P. putida* WT and *P. putida* pTT337, while in the presence of p-coumarate there is a 43-fold induction. The experimental protocol is shown in Supplementary Figure 1a and described in Methods. Statistically significant differences were determined using two-tailed Student's *t*-test (**, $P < 0.01$). Source data are provided as a Source Data file.

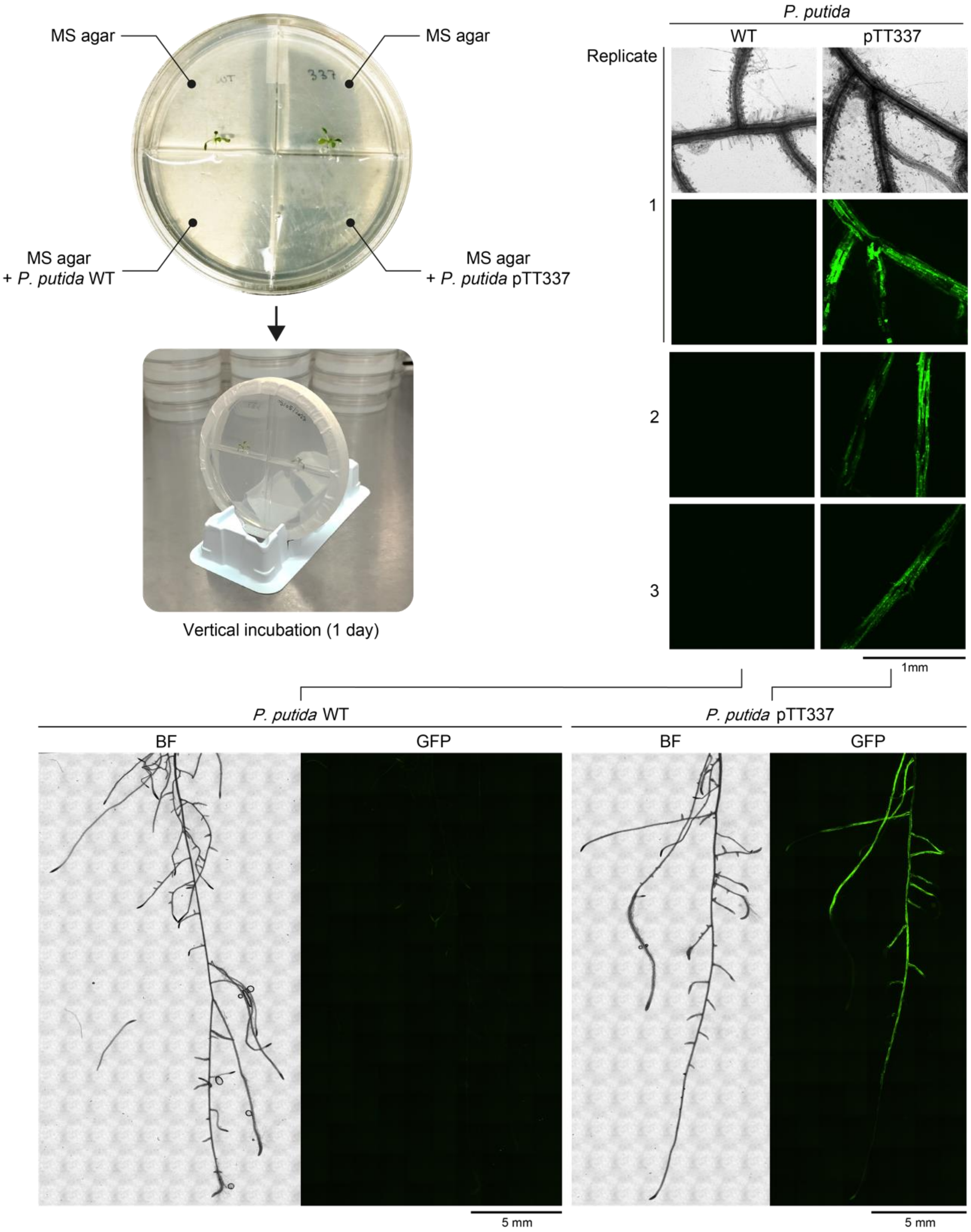

Supplementary Figure 21. Induction of the *A. thaliana* pC-HSL receiver by *P. putida* constitutively producing pC-HSL on solid media. The *A. thaliana* pC-HSL receiver (315_14_5_1) was grown for 7 days on MS agar before being transferred to a new MS agar plate containing either *P. putida* WT or *P. putida* carrying pTT337. The co-culture was grown for 24 hours before measuring GFP expression in the root using microscopy. Each row represents a replicate performed on different days with different plants. Images were taken with the A1R confocal microscope. A bright-field (BF) image is shown for the first replicate. Whole-root images are representative of experiments performed on three days with different plants. The experimental protocol is shown in Supplementary Figure 1b and described in Methods.

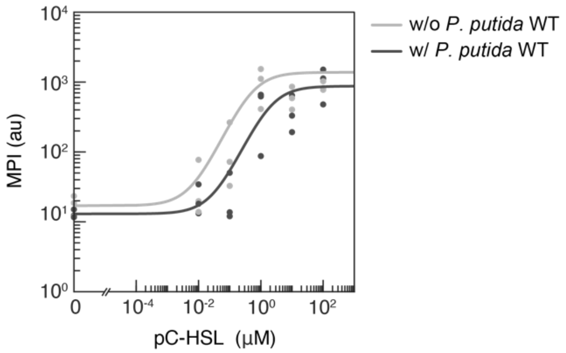

Supplementary Figure 22. The impact of bacteria on the response of the *A. thaliana* pC-HSL receiver. Data points were obtained for n = 3 experiments on different days. The lines are the fits of the means to Equation 1. Mean pixel intensity (MPI) was measured from images taken with the A1R confocal microscope. The experimental protocol is shown in Supplementary Figure 1a and described in Methods. Source data are provided as a Source Data file.

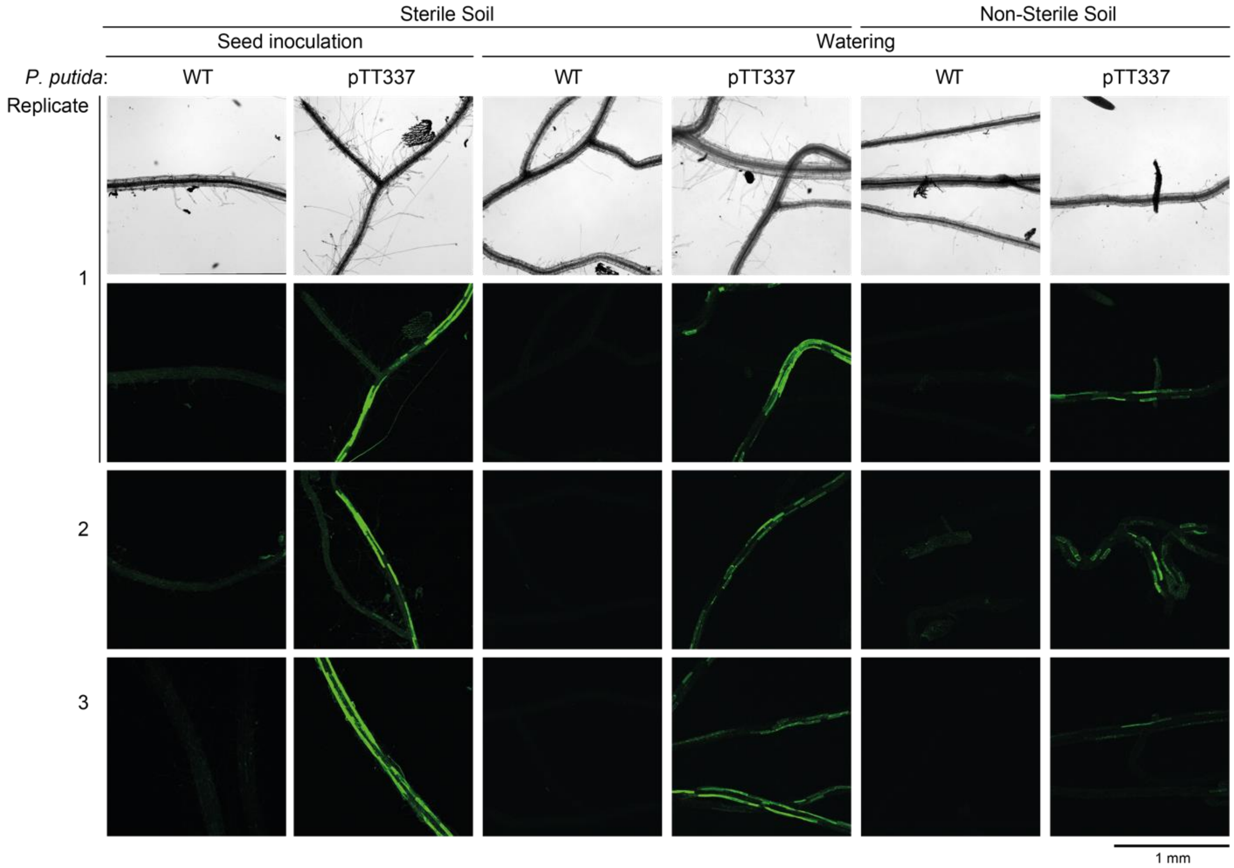

Supplementary Figure 23. Soil induction of the *A. thaliana* pC-HSL receiver by *P. putida* producing pC-HSL. The raw images of root tissue used to generate the data in Figure 3e, including statistics. Each row represents a replicate performed on different days with different plants (*A. thaliana* 315_14_5_1). Images were taken with the A1R confocal microscope. Bright-field (BF) images are provided for the first replicate. Whole-root images are shown for one replicate and are representative of experiments performed on three days with different plants. The experimental protocols are shown in Supplementary Figure 1d for seed inoculation and 1e for watering (Methods).

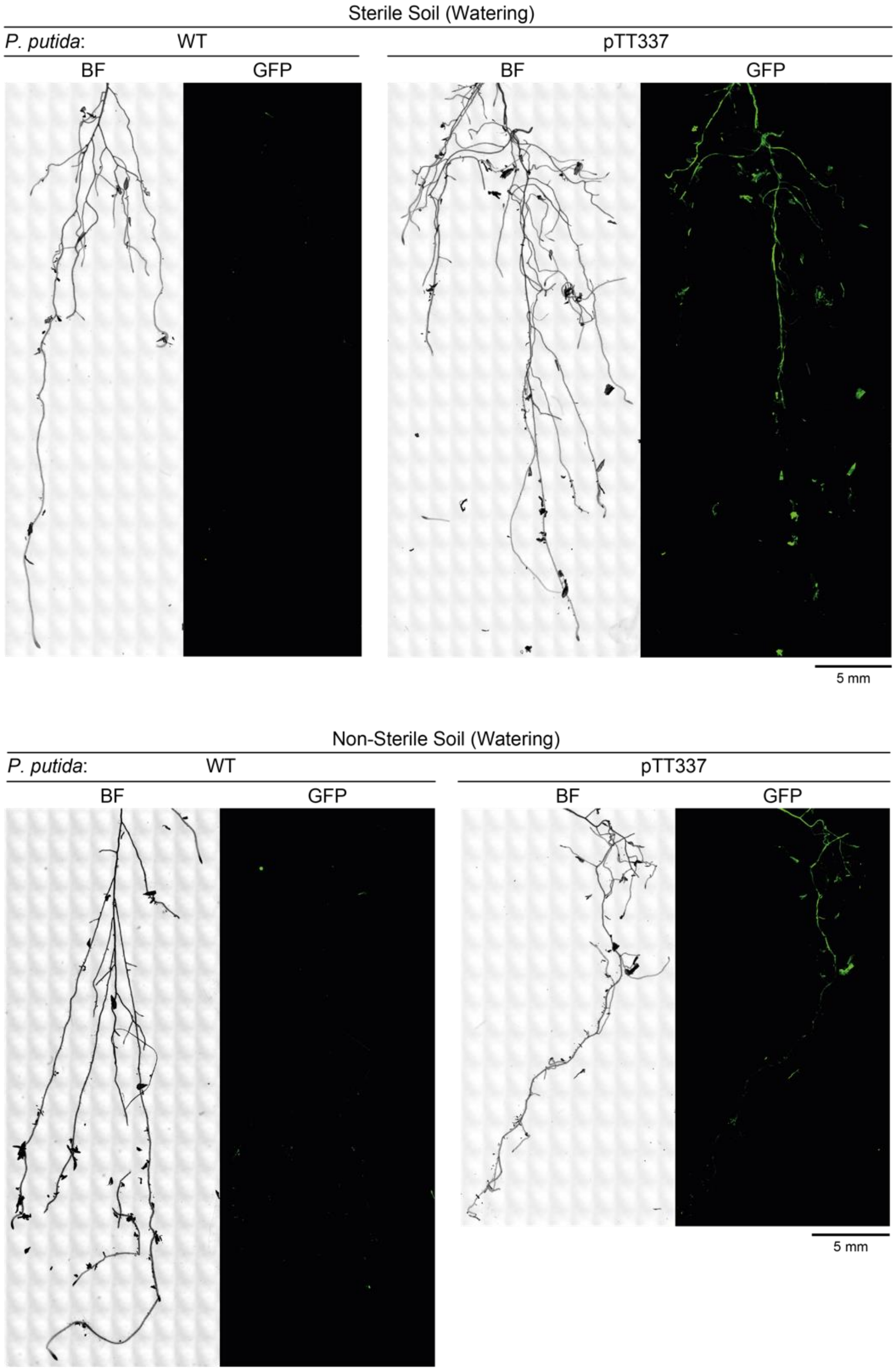

Supplementary Figure 24. Whole root images of the soil induction of the *A. thaliana* pC-HSL receiver by *P. putida* constitutively producing pC-HSL. Whole-root images of root tissue for the data plotted in Figure 3e and Supplementary Figure 22. Each row represents a replicate performed on different days with different plants (*A. thaliana* 315_14_5_1). Images were taken with the A1R confocal microscope. Bright-field (BF) and GFP images are shown. Whole-root images are shown for one replicate and are representative of experiments performed on three different days with different plants (*A. thaliana* 315_14_5_1). The experimental protocol is shown in Supplementary Figure 1e and described in Methods.

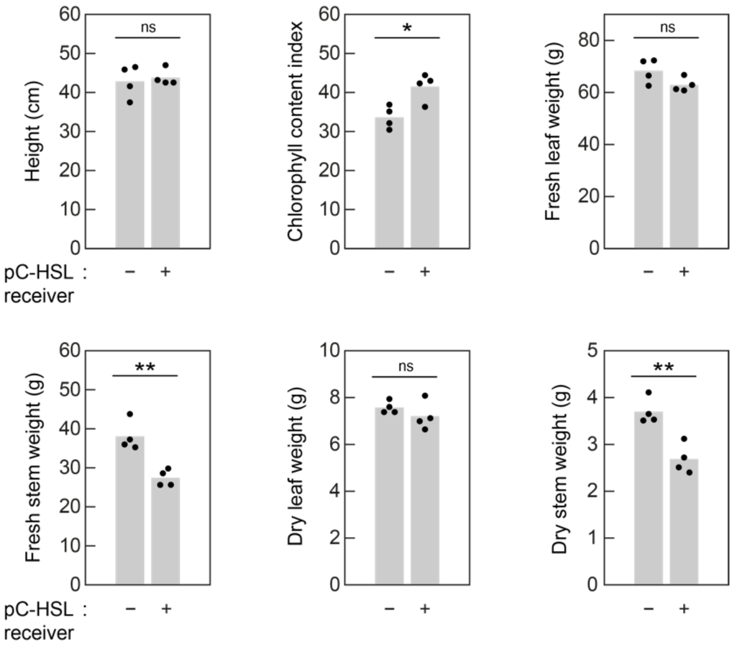

Supplementary Figure 25. Phenotypic analysis of *S. tuberosum* carrying the pC-HSL receiver. *S. tuberosum* wild-type (-) and containing the pC-HSL receiver (+) were compared (Methods). Data points were obtained for $n = 4$ plants and the bars represent the means of these points. Statistical significance was determined using two-tailed Student's *t*-test (ns, not significant $P > 0.05$; *, $P < 0.05$; **, $P < 0.01$). Source data are provided as a Source Data file.

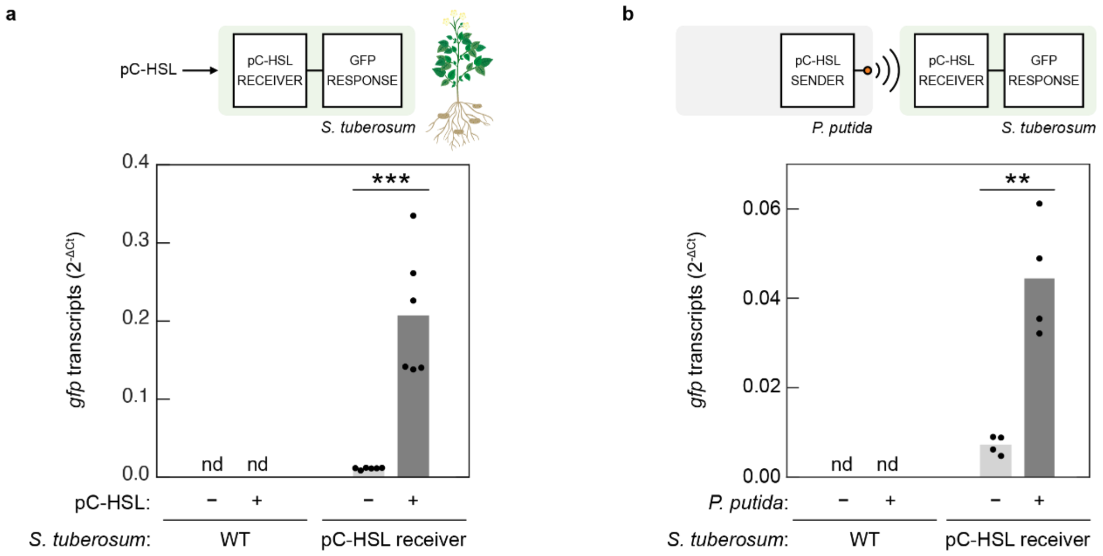

Supplementary Figure 26. Induction of the *S. tuberosum* pC-HSL receiver. a. *S. tuberosum* wild-type and the pC-HSL receiver in potato (*S. tuberosum* 315) were induced with 100 µM of pC-HSL. The transcription of *gfp* was determined through qRT-PCR performed on the RNA extracted from the roots of different plants (Methods). Data points represent the *gfp* transcripts for $n = 6$ plants and the bars represent the means. nd indicates that no transcripts were detected. **b.** The wild-type potato and the pC-HSL receiver in potato induced by *P. putida* constitutively producing pC-HSL (pTT337). The bacteria were co-cultured with the plant in the hydroponic system for 24 hours in MS:LB medium supplemented with 100 µM *p*-coumarate (Methods). The transcription of *gfp* was determined through qRT-PCR performed on the RNA extracted from the roots of different plants (Methods). Data points represent the *gfp* transcripts for $n = 4$ plants and the bars represent the means of these points. Statistical significance was determined using two-tailed Student's *t*-test (***, $P < 0.001$; **, $P < 0.01$). Source data are provided as a Source Data file.

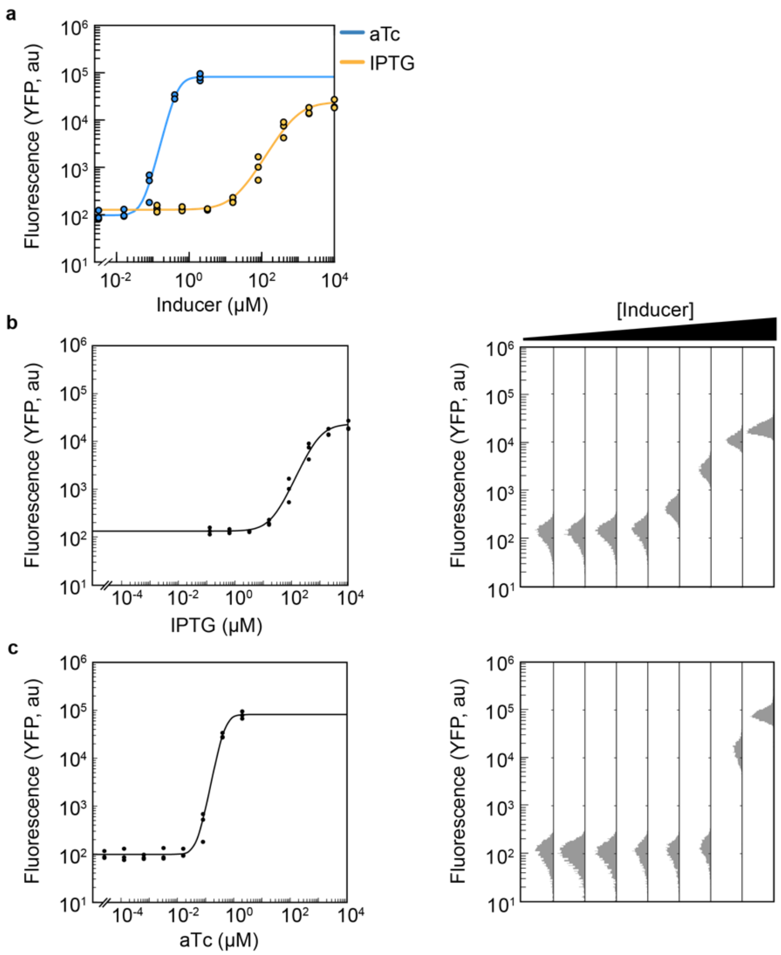

Supplementary Figure 27. Response functions for the sensors in *P. putida*. a. Response functions of *P. putida* pTT409 (IPTG sensor) and *P. putida* pTT410 (aTc sensor). The data were gathered for replicates performed on three days, the means of which were used to fit to Equation 1 (Supplementary Table 2). Representative histograms used to characterize the response functions are shown in **b** for *P. putida* pTT409 and in **c** for *P. putida* pTT410. From left to right in the cytometry plots, inducer concentrations were: 0.13, 0.64, 3.20, 16, 80, 400, 2000, 10000 µM IPTG; 0.0256, 0.128, 0.64, 3.2, 16, 80, 400, 2000 nM aTc. Source data are provided as a Source Data file.

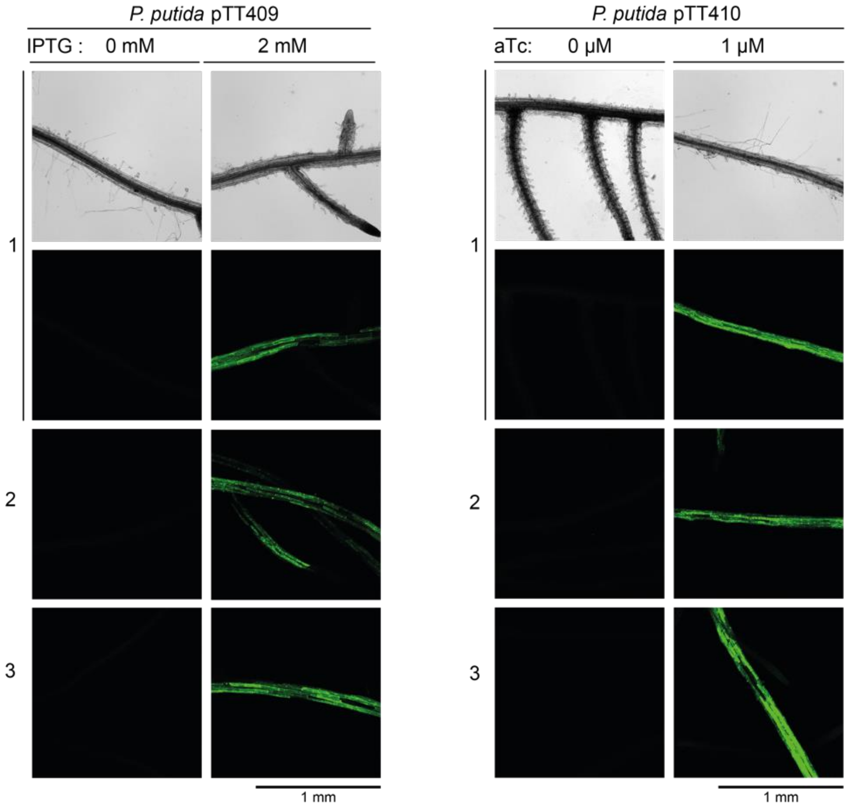

Supplementary Figure 28. The response of the *A. thaliana* pC-HSL receiver to the *P. putida* IPTG-aTc-induced production of pC-HSL. The raw images of root tissue used to generate the data in Figure 4a, including statistics. Images were taken with the A1R confocal microscope. Each row represents a replicate performed on different days with different plants (*A. thaliana* 315_14_5_1,). Bright-field (BF) images are provided for the first replicate. The experimental protocol is shown in Supplementary Figure 1a and described in Methods.

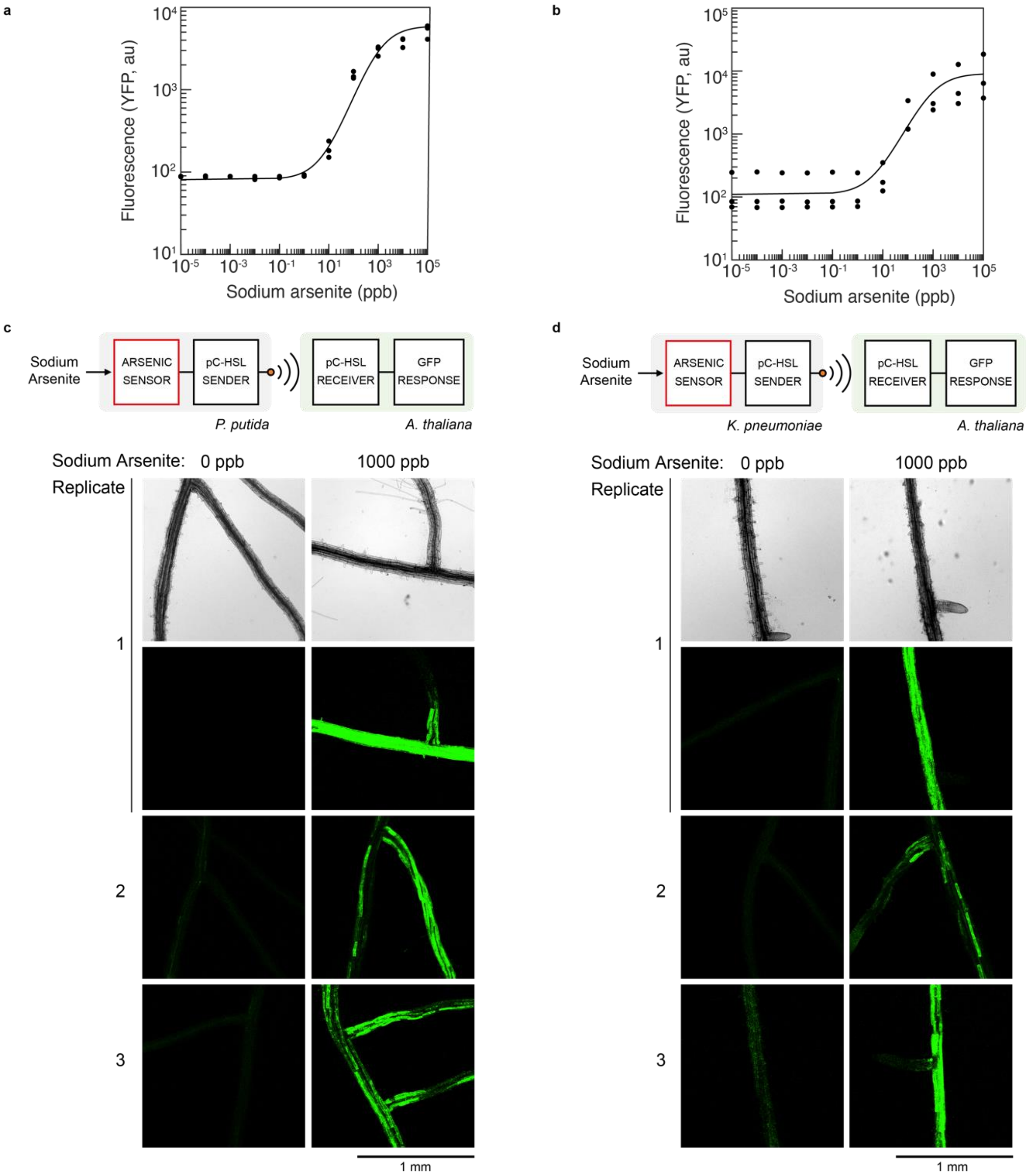

Supplementary Figure 29. Arsenic sensor response. a. Response function of *P. putida* pTT417 (arsenic sensor) and **b.** *K. pneumoniae* pTT417 (arsenic sensor). The data were gathered for replicates performed on three days, the means of which were used to fit to Equation 1 (parameters in Supplementary Table 2). **c.** The raw images of root tissue used to generate the data in Figure 4b, including statistics (inoculation with *P. putida*). **d.** The raw images of root tissue used to generate the data in Figure 4b, including statistics (inoculation with *K. pneumoniae*). For **c-d**, the concentrations of inducer used to induce the coculture was 1000 ppb. The experimental details are provided in Methods. Each row represents a replicate performed on different days with different plants (*A. thaliana* 315_14_5_1, Supplementary Table 3). Images were taken with the A1R confocal microscope. Bright-field and GFP images are provided for the first replicate. The experimental protocol is shown in Supplementary Figure 1a and described in Methods. Source data are provided as a Source Data file.

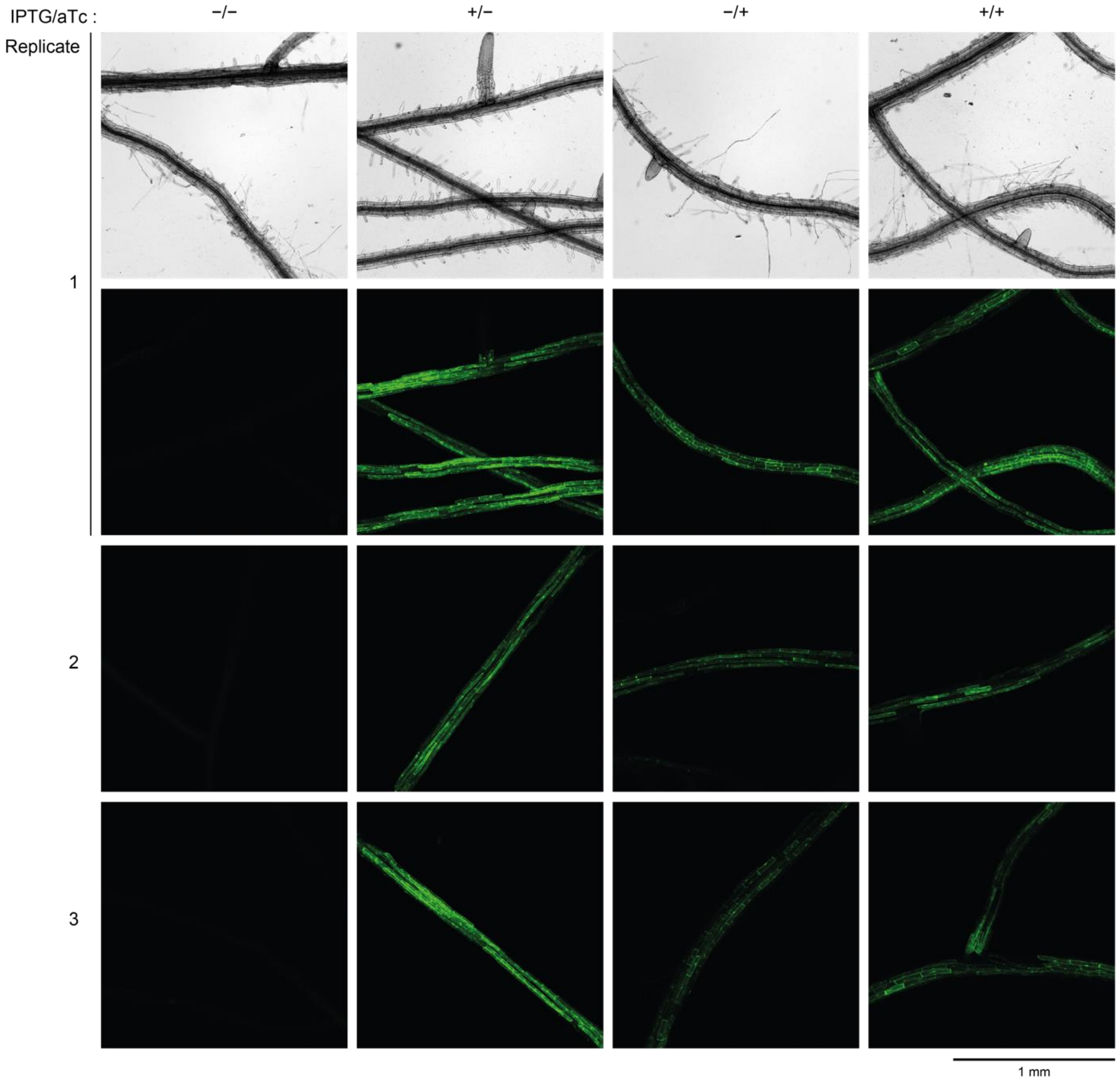

Supplementary Figure 30. Induction of the *A. thaliana* pC-HSL receiver by an OR gate in *P. putida* pC-HSL sender. The raw images of root tissue used to generate the data in Figure 4c are shown. The OR gate consists of *P. putida* sTT659 (Supplementary Table 4) containing the pTT434 plasmid (IPTG and aTc sensors controlling pC-HSL production, Supplementary Figure 34). The concentrations of inducers were 2 mM IPTG and 1 µM aTc. Each row represents a replicate performed on different days with different plants (*A. thaliana* 315_14_5_1). Images were taken with the A1R confocal microscope. Bright-field (BF) images are provided for the first replicate. The experimental protocol is shown in Supplementary Figure 1a and described in Methods.

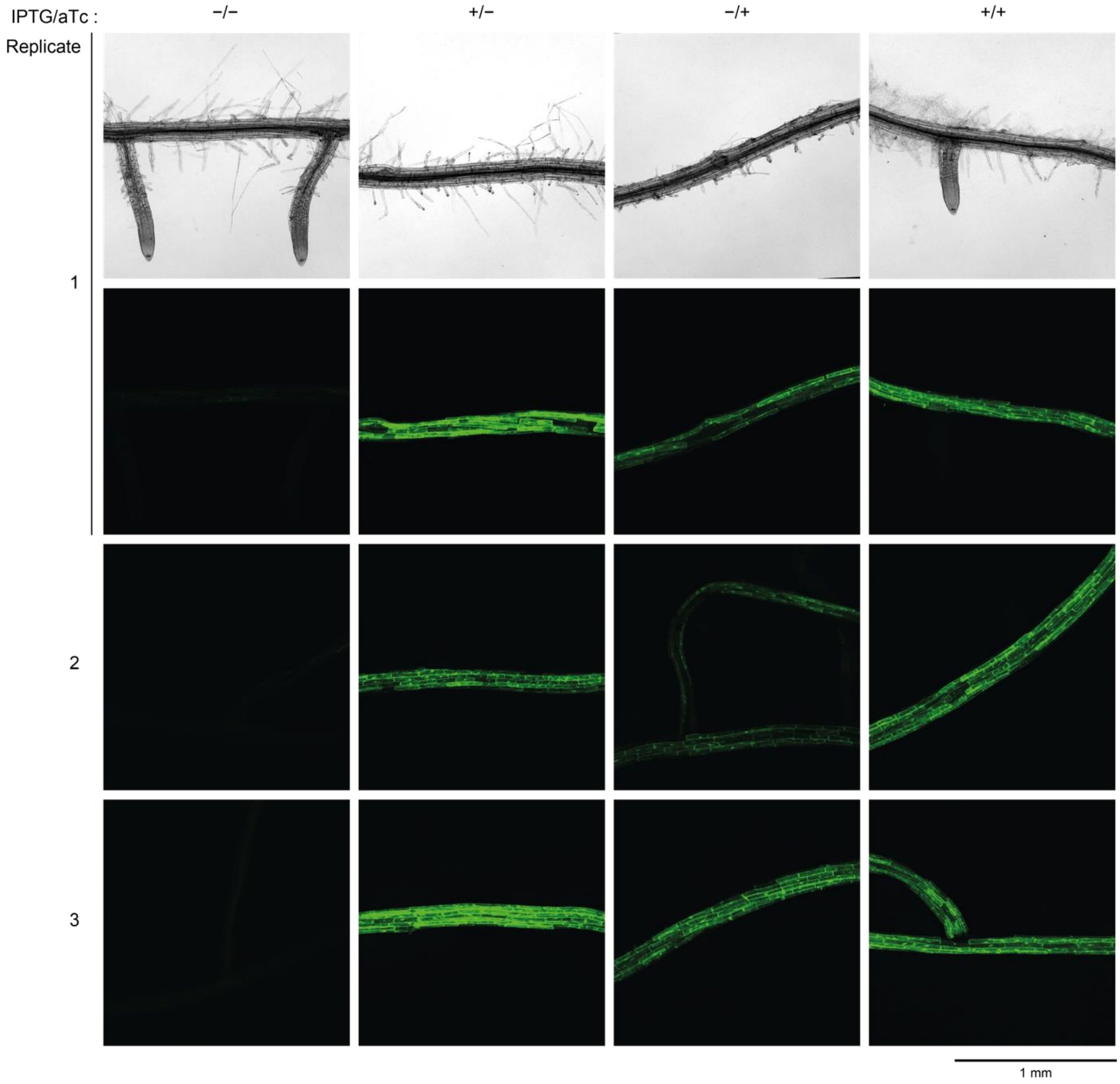

Supplementary Figure 31. Induction of the *A. thaliana* pC-HSL receiver by a consortium of *P. putida* pC-HSL senders. The raw images of root tissue used to generate the data in Figure 4c are shown. The consortium consists of *P. putida* pTT409 (IPTG sensor controlling pC-HSL production) and *P. putida* pTT410 (aTc sensor controlling pC-HSL production). The concentrations of inducers were 2 mM IPTG and 1 µM aTc. Each row represents a replicate performed on different days with different plants (*A. thaliana* 315_14_5_1). Images were taken with the A1R confocal microscope. Bright-field (BF) images are provided for the first replicate. The experimental protocol is shown in Supplementary Figure 1a and described in Methods.

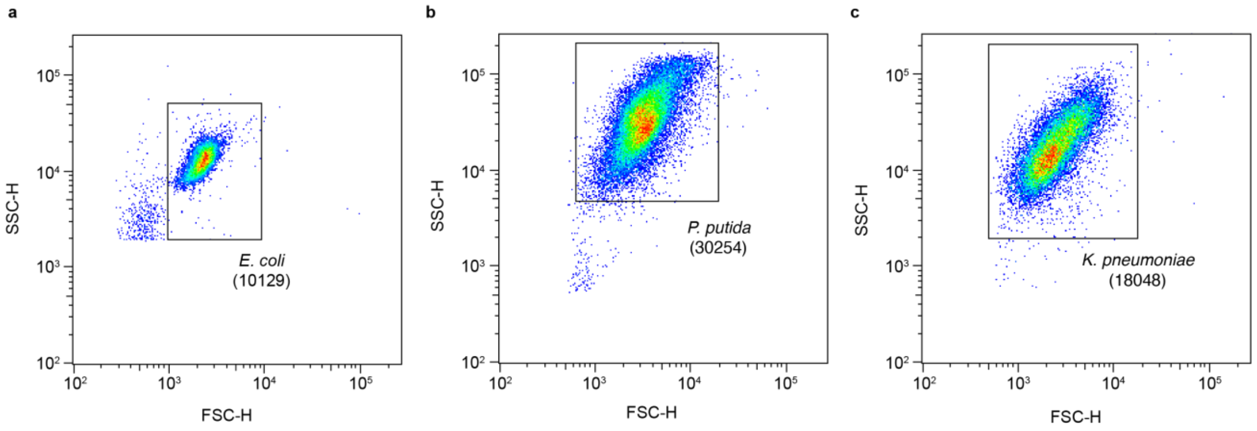

Supplementary Figure 32. Flow cytometry gating strategy. >10,000 events were collected, and gating was performed based on forward scatter (FSC-H) and side scatter (SSC-H) as indicated by the black rectangle: **a.** for *E. coli*: 2,000-50,000 SSC-H and 1,000-10,000 FSC-H; **b.** for *P. putida*: 5,000-200,000 SSC-H and 600-20,000 FSC-H; **c.** for *K. pneumoniae*: 2,000-200,000 SSC-H and 500-20,000 FSC-H.

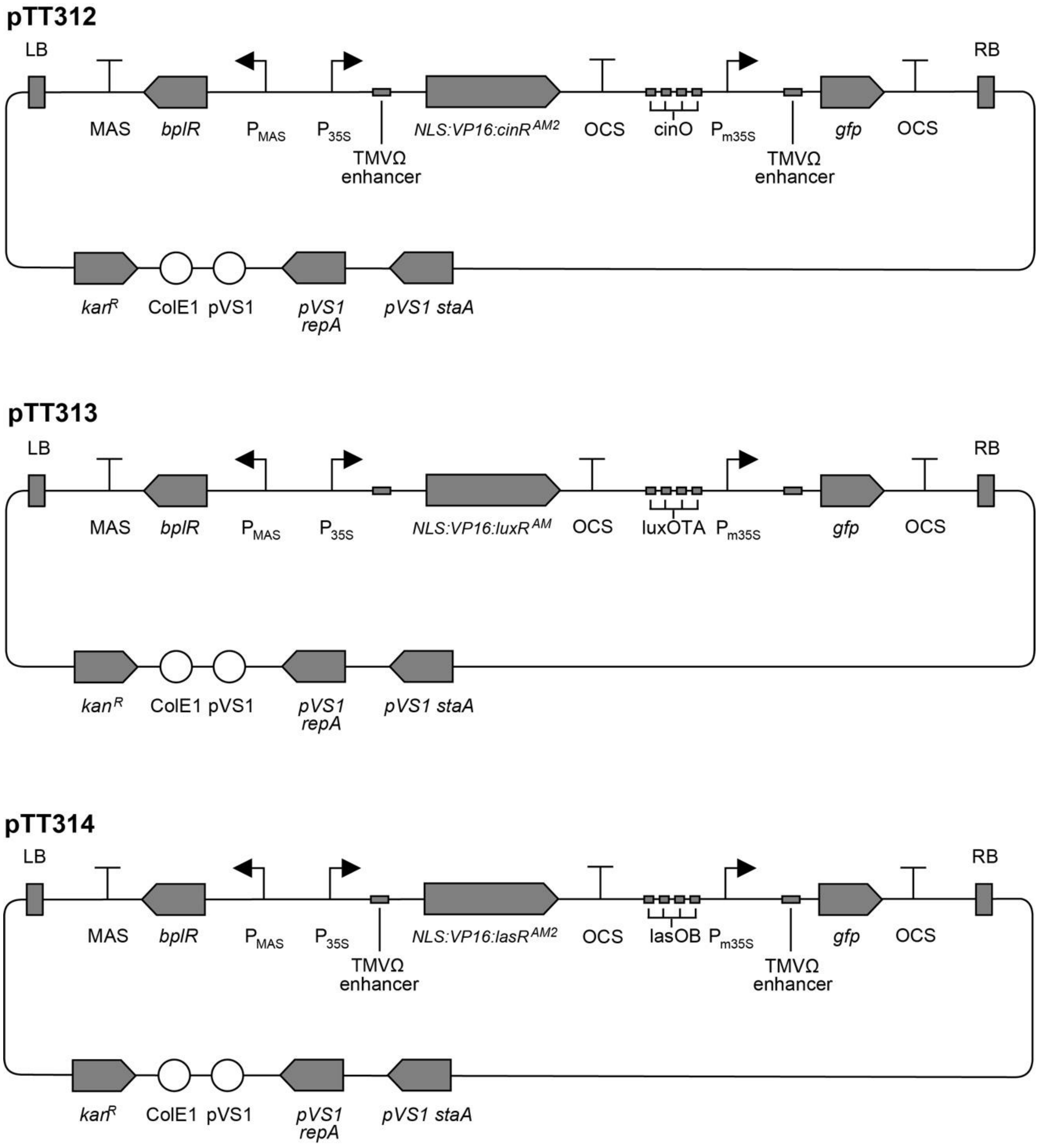

Supplementary Figure 33. Plasmid maps used to build *A. thaliana* lines containing HSL receivers. Plant lines generated with the above plasmids are recorded in Supplementary Table 3. The part sequences are provided in Supplementary Data 1 and full plasmid sequences are provided in Supplementary Data 3.

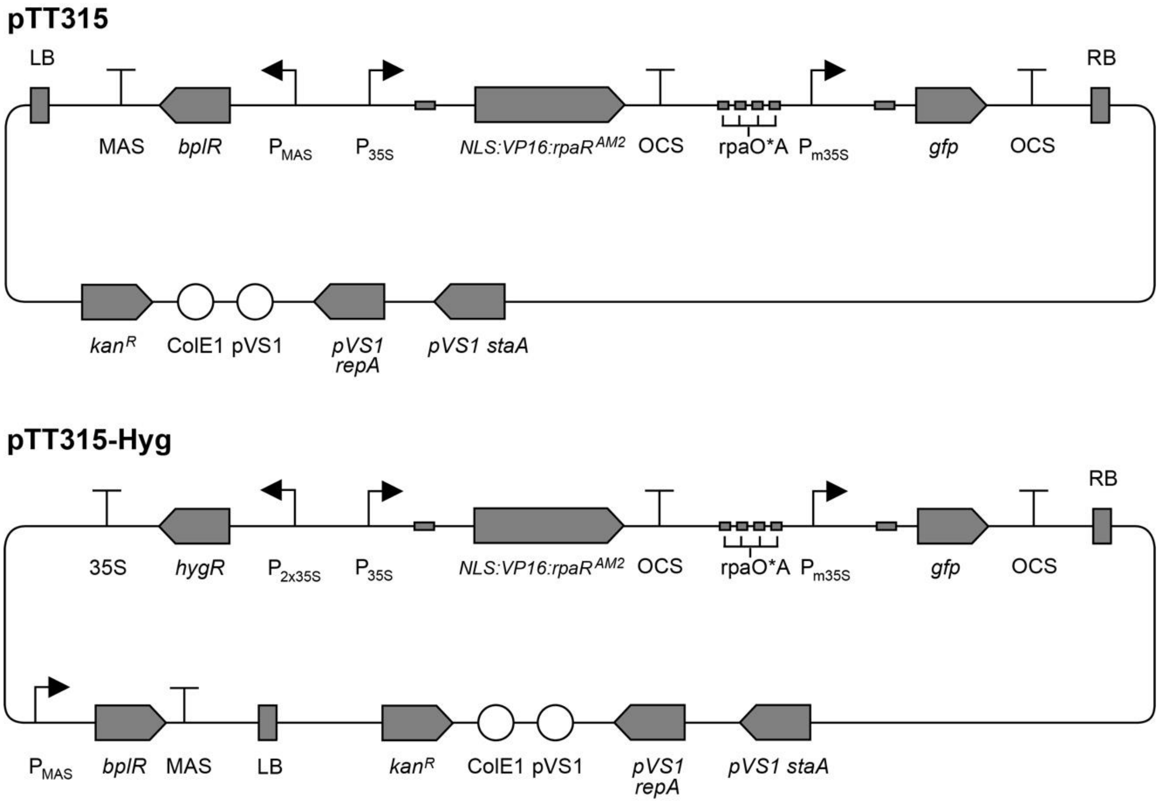

Supplementary Figure 34. Plasmid map used to build the *S. tuberosum* line containing the pC-HSL receiver. The plant line generated with the above plasmid is recorded in Supplementary Table 3. The part sequences are provided in Supplementary Data 1 and full plasmid sequences are provided in Supplementary Data 3.

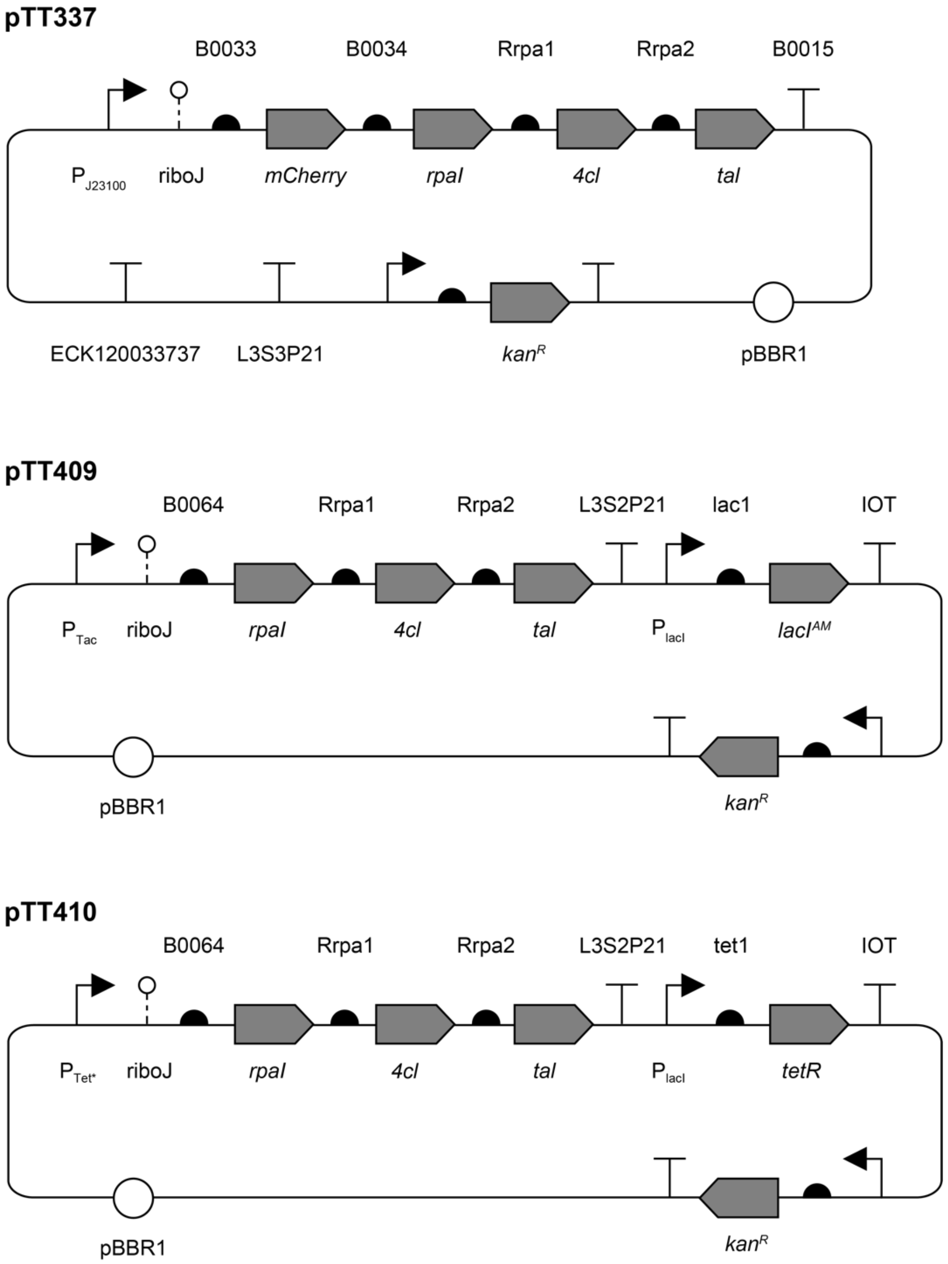

Supplementary Figure 35. Plasmids used for *P. putida* and *K. pneumoniae* for pC-HSL production. Parts sequences are provided in Supplementary Data 2 and full plasmid sequences are provided in Supplementary Data 3.

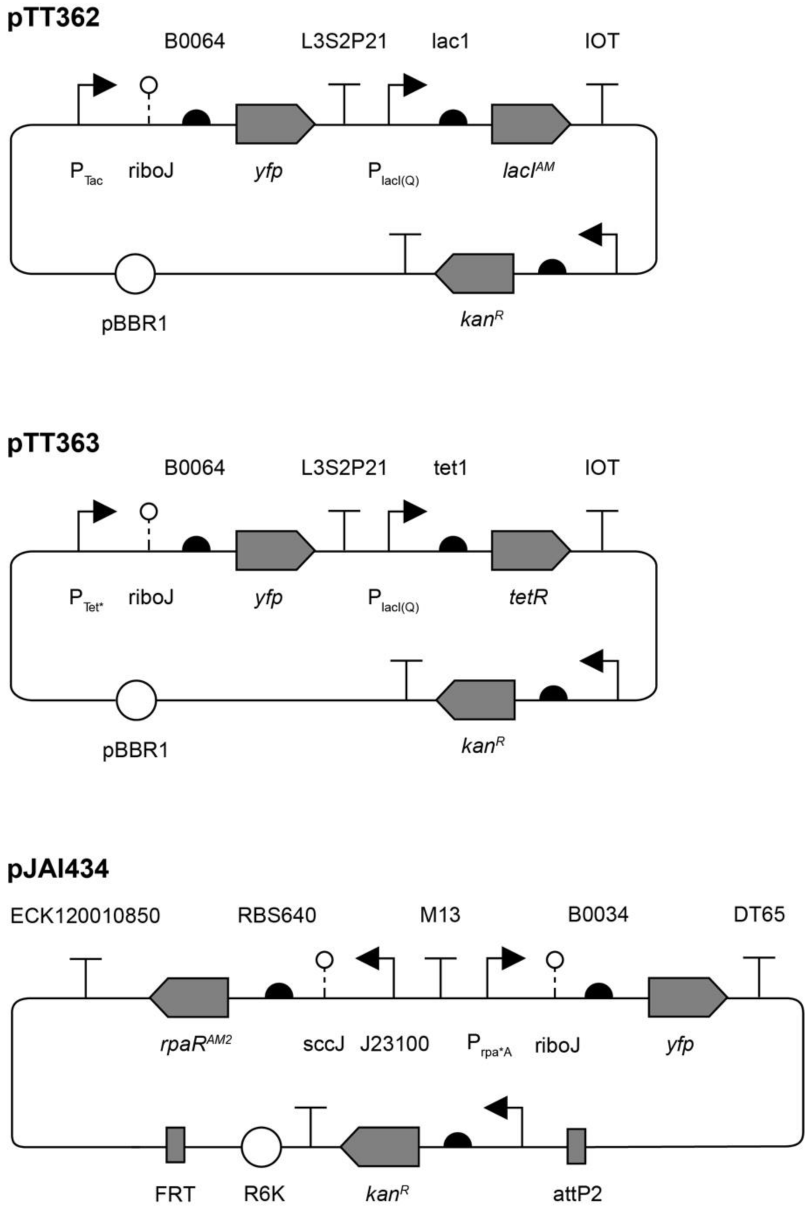

Supplementary Figure 36. Sensor plasmids used for *P. putida* and *E. coli*. Parts sequences are provided in Supplementary Data 2 and full plasmid sequences are provided in Supplementary Data 3.

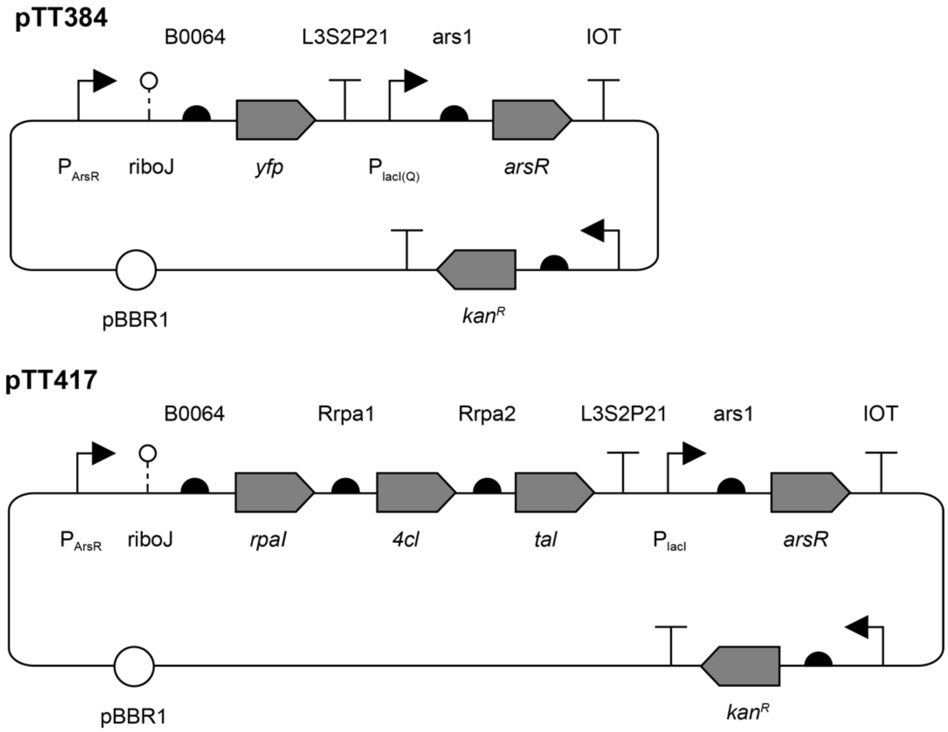

Supplementary Figure 37. Arsenic sensor plasmids used for *P. putida* and *E. coli*. Parts sequences are provided in Supplementary Data 2 and full plasmid sequences are provided in Supplementary Data 3.

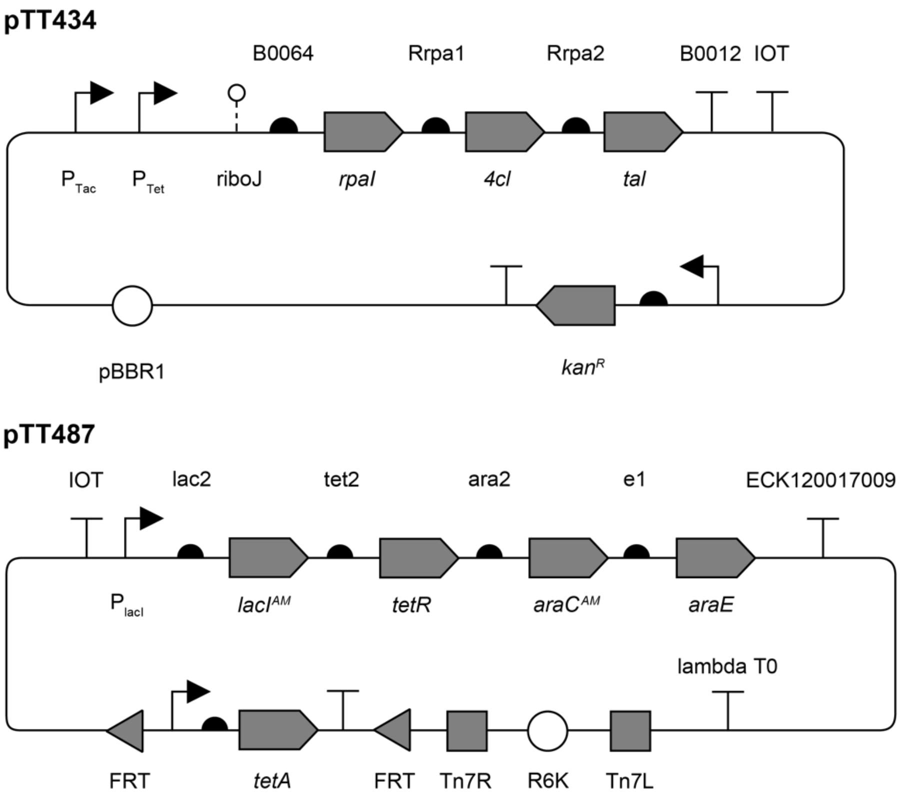

Supplementary Figure 38. Plasmids used for building the OR gate in *P. putida*. Parts sequences are provided in Supplementary Data 2 and full plasmid sequences are provided in Supplementary Data 3.

Supplementary Table 1. Responses of sensors in *A. thaliana*.

	Parameters[a]			
Sensor	κ (µM)	*n*	y_{min} (au)	y_{max} (au)
pC-HSL	0.1	2.9	5.4	870
OC12-HSL	7.2	1.5	5.8	230
pC-HSL	0.5	1.0	17	1400

[a]Parameters are fits to Equation **1**.

Supplementary Table 2. Responses of sensors in bacteria.

	Species	Parameters[a]			
Sensor		κ	n	y_{min} (au)	y_{max} (au)
pC-HSL	*E. coli*	0.006 µM	1.6	510	110000
IPTG	*P. putida*	950 µM	1.5	130	19000
aTc	*P. putida*	0.5 µM	3.7	120	96000
Sodium arsenite	*P. putida*	11000 ppb	0.8	80	6000
Sodium arsenite	*K. pneumoniae*	10000 ppb	0.8	120	10000

[a]Parameters are fits to Equation **1**.

Supplementary Table 3. Plant lines used in this study.

Line	Source	Resistance	Description
Arabidopsis thaliana Col-0	ABRC CS70000	-	-
Solanum tuberosum	Ref [1]	-	-
A. thaliana Col-0 312_7_5	This study	Phosphinothricin	T3 line generated by pTT312
A. thaliana Col-0 313_3_4	This study	Phosphinothricin	T3 line generated by pTT313
A. thaliana Col-0 314_12_2	This study	Phosphinothricin	T3 line generated by pTT314
A. thaliana Col-0 315_4_2	This study	Phosphinothricin	T3 line generated by pTT315
A. thaliana Col-0 315_7_2	This study	Phosphinothricin	T3 line generated by pTT315
A. thaliana Col-0 315_11_2	This study	Phosphinothricin	T3 line generated by pTT315
A. thaliana Col-0 315_14_4	This study	Phosphinothricin	T3 line generated by pTT315
A. thaliana Col-0 315_14_5	This study	Phosphinothricin	T3 line generated by pTT315
A. thaliana Col-0 315_17_2	This study	Phosphinothricin	T3 line generated by pTT315
A. thaliana Col-0 315_22_2	This study	Phosphinothricin	T3 line generated by pTT315
A. thaliana Col-0 315_23_2	This study	Phosphinothricin	T3 line generated by pTT315
A. thaliana Col-0 315_24_3	This study	Phosphinothricin	T3 line generated by pTT315
A. thaliana Col-0 315_14_5_1	This study	Phosphinothricin	T4 line generated by pTT315
S. tuberosum 315	This study	Hygromycin	Generated by pTT315-Hyg

Plasmid maps of plasmids transformed in *A. tumefaciens* for *A. thaliana* and *S. solanum* transformation are shown in Supplementary Figures 33 and 34.

Supplementary Table 4. Bacterial strains used in this study.

Strain	Source	Description[a]
Escherichia coli NEB 10β	NEB C3019I	
Pseudomonas putida KT2440	ATCC 47054	
Agrobacterium tumefaciens GV3101	Gold Bio GV3101	
Escherichia coli MG1655 YJP_MKC173	Ref [2]	
Escherichia coli MG1655 sTT658	This study	*E. coli* MG1655 YJP_MKC173 with $RpaR^{AM}$ and P_{rpa*A}-YFP genomically integrated at the LP1 landing pad locus using the pJAI434 plasmid
Pseudomonas putida sTT659	This study	*Pseudomonas putida* KT2440::Tn7-tcR-lacI-tetR-araCE inserted into the attTn7 site using the pTT487 plasmid

[a]Plasmid maps of pJAI434 and pTT487 are shown in Supplementary Figure 36 and 38 respectively.

Supplementary references

1. Occhialini, A. et al. Mini-synplastomes for plastid genetic engineering. *Plant Biotechnology Journal* **20**, 360-373 (2022).
2. Park, Y., Espah Borujeni, A., Gorochowski, T.E., Shin, J. & Voigt, C.A. Precision design of stable genetic circuits carried in highly-insulated *E. coli* genomic landing pads. *Mol Syst Biol* **16**, e9584 (2020).
