# Supplementary material for: Synthetic microbe-to-plant communication channels
Source: Nat Commun. 2024 Feb 28;15:1817. doi: 10.1038/s41467-024-45897-6 (PMC10901793; doi:10.1038/s41467-024-45897-6)
Supplement: Supplementary file 3 — Description of Additional Supplementary Files [file 41467_2024_45897_MOESM3_ESM.pdf]

Description of Additional Supplementary Files

File: Supplementary Data 1
Description: Plant genetic parts

File: Supplementary Data 2
Description: Bacterial genetic parts

File: Supplementary Data 3
Description: Plasmid sequences

File: Supplementary Movie 1
Description: Root hair induction of the *A. thaliana* pC-HSL receiver by pC-HSL in the hydroponic system.
Video of the 3D reconstruction of uninduced roots (replicate 1) from Supplementary Figure 9.

File: Supplementary Movie 2
Description: Video of the 3D reconstruction of uninduced roots (replicate 2) from Supplementary Figure 9.

File: Supplementary Movie 3
Description: Video of the 3D reconstruction of induced roots (replicate 1) from Supplementary Figure 9.

File: Supplementary Movie 4
Description: Video of the 3D reconstruction of induced roots (replicate 2) from Supplementary Figure 9.
