# Supplementary material for: Synthetic microbe-to-plant communication channels
Source: Nat Commun. 2024 Feb 28;15:1817. doi: 10.1038/s41467-024-45897-6 (PMC10901793; doi:10.1038/s41467-024-45897-6)
Supplement: Supplementary file 11 — Reporting Summary [file 41467_2024_45897_MOESM11_ESM.pdf]

nature portfolio

Corresponding author(s): Chritopher A. Voigt

Last updated by author(s): 02/06/2024

Reporting Summary

Nature Portfolio wishes to improve the reproducibility of the work that we publish. This form provides structure for consistency and transparency in reporting. For further information on Nature Portfolio policies, see our Editorial Policies and the Editorial Policy Checklist.

Statistics

For all statistical analyses, confirm that the following items are present in the figure legend, table legend, main text, or Methods section.

n/a	Confirmed	
☐	☒	The exact sample size (*n*) for each experimental group/condition, given as a discrete number and unit of measurement
☐	☒	A statement on whether measurements were taken from distinct samples or whether the same sample was measured repeatedly
☐	☒	The statistical test(s) used AND whether they are one- or two-sided<br>*Only common tests should be described solely by name; describe more complex techniques in the Methods section.*
☒	☐	A description of all covariates tested
☒	☐	A description of any assumptions or corrections, such as tests of normality and adjustment for multiple comparisons
☐	☒	A full description of the statistical parameters including central tendency (e.g. means) or other basic estimates (e.g. regression coefficient) AND variation (e.g. standard deviation) or associated estimates of uncertainty (e.g. confidence intervals)
☐	☒	For null hypothesis testing, the test statistic (e.g. *F*, *t*, *r*) with confidence intervals, effect sizes, degrees of freedom and *P* value noted<br>*Give P values as exact values whenever suitable.*
☒	☐	For Bayesian analysis, information on the choice of priors and Markov chain Monte Carlo settings
☒	☐	For hierarchical and complex designs, identification of the appropriate level for tests and full reporting of outcomes
☒	☐	Estimates of effect sizes (e.g. Cohen's *d*, Pearson's *r*), indicating how they were calculated

Our web collection on statistics for biologists contains articles on many of the points above.

Software and code

Policy information about availability of computer code

Data collection	[flow] BD LSR Fortessa with BD FACSDiva 8.0; [microscope] Nikon Ti-E fluorescence microscope with NIS-Elements 4.0; Leica SP8 fluorescence microscope with LAS X 1.4.5; [geldoc imager] Bio-Rad ChemiDocMP Imaging system #1708280 with Image Lab 4.0; [qPCR] Thermo Fisher Scientific QuantStudio 3 Real-Time PCR System with Design and Analysis software v.1.5.1.
Data analysis	[image processing] images were analyzed with ImageJ2 v.2.9.0 and a custom script available at https://github.com/VoigtLab/plant-microbe-communication; [flow cytometry] gating and standard reporter level calculation was done using FlowJo 7.6 and Cytoflow v.1.2; [qPCR] Microsoft Excel for Mac 16.70; [graphics] Microsoft Excel for Mac 16.70, MATLAB R2021a, Adobe Illustrator 2023.

For manuscripts utilizing custom algorithms or software that are central to the research but not yet described in published literature, software must be made available to editors and reviewers. We strongly encourage code deposition in a community repository (e.g. GitHub). See the Nature Portfolio guidelines for submitting code & software for further information.

Data

Policy information about availability of data

All manuscripts must include a data availability statement. This statement should provide the following information, where applicable:

- Accession codes, unique identifiers, or web links for publicly available datasets
- A description of any restrictions on data availability
- For clinical datasets or third party data, please ensure that the statement adheres to our policy

The P. putida KT2440 (Taxonomy ID: 160488) and K. pneumoniae 342 (Taxonomy ID: 507522) genomes were used to search for homologues of luxI (Vibrio fischeri, NCBI-Protein ID: AAW87994), cinI (Rhizobium leguminosarum, NCBI-Protein ID: WP_018242930), lasI (Pseudomonas aeruginosa, NCBI-Protein ID: QPV56976) and rpaI (Rhodopseudomonas palustris, NCBI-Protein ID: WBU30219) query genes using the protein basic local alignment search tool (BLASTp; blast.ncvi.nlm.nih.gov) with the blastp (protein-protein BLAST) program and default parameters. Plant genetic parts are available as part of Supplementary Data 1 "plant-genetic-parts.xlsx". Bacterial genetic parts are available as part of Supplementary Data 2 "bacteria-genetic-parts.xlsx". Plasmid maps are available as part of Supplementary Data 3 "plasmid-sequences.xlsx". Movie files from Supplementary Figure 9 are available as Supplementary Movies 1-4. The data that support the findings of this study are available with the main text and Supplementary Information. All microscopy images used for fluorescence quantification are available in the Supplementary Information at a resolution of 600 dpi and the raw TIFF files are available at: https://doi.org/10.5281/zenodo.10601326. Source Data are provided with this paper.

Research involving human participants, their data, or biological material

Policy information about studies with human participants or human data. See also policy information about sex, gender (identity/presentation), and sexual orientation and race, ethnicity and racism.

Reporting on sex and gender	N/A
Reporting on race, ethnicity, or other socially relevant groupings	N/A
Population characteristics	N/A
Recruitment	N/A
Ethics oversight	N/A

Note that full information on the approval of the study protocol must also be provided in the manuscript.

Field-specific reporting

Please select the one below that is the best fit for your research. If you are not sure, read the appropriate sections before making your selection.

☒ Life sciences ☐ Behavioural & social sciences ☐ Ecological, evolutionary & environmental sciences

For a reference copy of the document with all sections, see nature.com/documents/nr-reporting-summary-flat.pdf

Life sciences study design

All studies must disclose on these points even when the disclosure is negative.

Sample size	No statistical methods were used to predetermine sample size. At least three biological replicates were performed on separate days for all experiments. Three replicates was sufficient for consistent and reproducible results to support claims made in this paper.
Data exclusions	All data was included.
Replication	All experimental claims have been tested at least three times on different days, following the reported methods. Results were repeatable across replicates.
Randomization	Randomization was not relevant for our study as both control and experimental samples were derived from T3 homozygous lines, and treatments were applied using bacterial cultures of the same genetic background, ensuring uniformity and focusing on the treatment effects.
Blinding	Blinding was not applicable for our study as data collection utilized objective methods like flow cytometry and qPCR, and automated ImageJ thresholding minimized bias in microscopy analysis. While investigators were not blinded during sample collection, the uniform application of automatic thresholding for fluorescence quantification of all samples controlled for potential observer bias.

Reporting for specific materials, systems and methods

We require information from authors about some types of materials, experimental systems and methods used in many studies. Here, indicate whether each material, system or method listed is relevant to your study. If you are not sure if a list item applies to your research, read the appropriate section before selecting a response.

Materials & experimental systems

n/a	Involved in the study
☒	☐ Antibodies
☒	☐ Eukaryotic cell lines
☒	☐ Palaeontology and archaeology
☒	☐ Animals and other organisms
☒	☐ Clinical data
☒	☐ Dual use research of concern
☐	☒ Plants

Methods

n/a	Involved in the study
☒	☐ ChIP-seq
☐	☒ Flow cytometry
☒	☐ MRI-based neuroimaging

Dual use research of concern

Policy information about dual use research of concern

Hazards

Could the accidental, deliberate or reckless misuse of agents or technologies generated in the work, or the application of information presented in the manuscript, pose a threat to:

No	Yes
☒	☐ Public health
☒	☐ National security
☒	☐ Crops and/or livestock
☒	☐ Ecosystems
☒	☐ Any other significant area

Experiments of concern

Does the work involve any of these experiments of concern:

No	Yes
☒	☐ Demonstrate how to render a vaccine ineffective
☒	☐ Confer resistance to therapeutically useful antibiotics or antiviral agents
☒	☐ Enhance the virulence of a pathogen or render a nonpathogen virulent
☒	☐ Increase transmissibility of a pathogen
☒	☐ Alter the host range of a pathogen
☒	☐ Enable evasion of diagnostic/detection modalities
☒	☐ Enable the weaponization of a biological agent or toxin
☒	☐ Any other potentially harmful combination of experiments and agents

Flow Cytometry

Plots

Confirm that:

☒ The axis labels state the marker and fluorochrome used (e.g. CD4-FITC).

☒ The axis scales are clearly visible. Include numbers along axes only for bottom left plot of group (a 'group' is an analysis of identical markers).

☒ All plots are contour plots with outliers or pseudocolor plots.

☒ A numerical value for number of cells or percentage (with statistics) is provided.

Methodology

Sample preparation: Bacterial cells were diluted into PBS before running into the flow cytometer.

Instrument	BD LSR Fortessa
Software	Data was collected using FACS Diva software 8.0, FlowJo 7.6 and Cytoflow v.1.2 were used for data anaylsis.
Cell population abundance	10,000 gated cells per sample.
Gating strategy	The following gating strategy was used to separate the cells from debris: for P. putida: 5,000-50,000 SSC-H and 1,000-12,000 FSC-H; for E. coli: 2,000-50,000 SSC-H and 1,000-10,000 FSC-H; for K. pneumoniae: 2,000-200,000 SSC-H and 500-20,000 FSC-H.

☒ Tick this box to confirm that a figure exemplifying the gating strategy is provided in the Supplementary Information.
